# Unveiling gender disparities in corporate board career paths using deep learning

**DOI:** 10.1016/j.patter.2026.101495

**Published:** 2026-03-12

**Authors:** Yuhao Zhou, Wenhao Chen, María Óskarsdóttir, Matt Davison, Cristián Bravo

**Affiliations:** 1 Department of Statistical and Actuarial Sciences, Western University, London, ON N6A 5B7, Canada; 2 School of Mathematical Sciences, University of Southampton, Southampton SO17 1BJ, UK; 3 Department of Computer Science, Reykjavik University, 102 Reykjavík, Iceland

**Keywords:** corporate governance, social networks, gender equality, career trajectories, deep learning

## Abstract

In this study, we investigate the relationship between professional networks and gender disparities in corporate board appointments, focusing on publicly traded Canadian companies. Using data from over 19,000 senior managers and board members in more than 700 firms from 2000 to 2022, we combine social network analysis with a causal learning framework and long short-term memory (LSTM) models to examine how networks act as both enablers and barriers to achieving gender diversity in leadership. Our findings highlight a clear glass-ceiling effect: female board members must build wider and more influential networks than men to reach similar positions of influence, even when their demographics and career paths are comparable. Gender-specific personalized PageRank further reveals the strong role of female-to-female connections in supporting women’s advancement. This research contributes to a broader discussion of corporate governance and gender diversity, highlighting the need for inclusive networking and mentorship initiatives to reduce existing barriers.

## Highlights

• Women need higher network centrality than men to attain similar board roles
• Female-to-female professional ties strongly boost women’s chances of promotion
• Career-path sequences and networks jointly reveal gendered opportunity gaps
• Controlled LSTM models show the networks’ importance in realizing board access

## The bigger picture

Corporate boards play a central role in shaping business strategy, executive pay, and long-term economic outcomes. Despite decades of public attention and policy debate, women remain underrepresented in these powerful decision-making bodies. Discussions about this gap often focus on individual qualifications, career choices, or formal diversity rules. Less attention is paid to the informal social processes that influence who is noticed, trusted, and ultimately invited into leadership roles. Professional relationships formed over the course of a career, for example, through shared academic training at the same institutions, workplaces, collaborations, and mentorship, are a major pathway into senior positions. These networks are not simply a record of past experience; they actively shape access to information, endorsements, and opportunities. When leadership selection relies heavily on such relationships, even subtle differences in how networks are built and valued can produce lasting inequality.

This work highlights how gender disparities can persist even when men and women have similar experience and career histories. Women who attain board positions tend to have unusually broad and influential professional connections, suggesting that they must clear a higher informal threshold to reach the same roles as men. At the same time, connections among senior women emerge as an important source of support, pointing to the value of mentorship and sponsorship within underrepresented groups. These disparities point to a structural form of disadvantage rooted in the professional network itself, thereby shifting the attention from individual shortcomings to organizational practices. It underscores the importance of widening recruitment channels, reducing overreliance on closed networks, and actively fostering inclusive professional relationships. Addressing these hidden structural barriers is essential for building more equitable and effective corporate leadership.

## Teaser

Gender gaps in corporate boardrooms are shaped not only by hiring decisions but also by the structure of professional networks. Using 23 years of career histories and network data on thousands of executives, we show in this study that women must hold broader and more central positions than men to reach similar board roles. Connections to other female leaders are especially important. These findings illustrate how everyday networking practices can help reduce or reinforce inequality in corporate leadership.

## Introduction

During the past decade, efforts to increase gender diversity in corporate boardrooms have made significant progress. Studies have correlated a diverse board composition with significant benefits, including lower financial risks,^1^^,^^2^ lower borrowing costs,^3^^,^^4^ and improved financial performance.^5^^,^^6^ However, the board member nomination and selection process often lacks transparency, differing greatly from other labor markets in which supply and demand meet in open exchanges.^7^

Typically, current board members utilize their personal networks to recruit new directors,^8^^,^^9^ a practice that differs from competitive recruitment norms.^7^ Although professional networks are vital for career progression,^10^^,^^11^ reliance on them can perpetuate existing inequalities. Research indicates that “old boys” networks and closed circles significantly shape board composition,^12^ often resulting in lower market values and higher financial misrepresentation for firms with insulated boards.^13^ Furthermore, social network data can subtly but powerfully influence managers’ perceptions during selection,^14^ creating a feedback loop that disadvantages outsiders.

This network-driven dynamic creates specific barriers for women. Gender stereotypes continue to bias professional advancement,^15^ and the benefits of networking are not uniformly experienced across genders. For example, men tend to gain more from larger networks, whereas women’s networks often differ significantly in terms of size, strength, and access to influential figures.^16^^,^^17^^,^^18^ Consequently, while gender diversity is crucial for effective corporate governance,^19^^,^^20^ its realization is often obstructed by equitable access to these influential circles. Despite a large body of literature exploring these dynamics in corporate governance,^21^^,^^22^ relatively few studies have systematically combined career trajectory with network analysis to understand these disparities.

Methodologically, previous research in this domain has primarily employed conventional statistical methods, such as regression analysis.^7^^,^^12^ Although useful, modern methods are able to capture complex, nonlinear dynamics of professional ties and career sequences. Several researchers, including us, have successfully applied the combination of machine learning (ML) and network science in adjacent fields, such as fraud detection and credit risk, replacing regression with ML models. Doing so has resulted in significant performance gains,^23^^,^^24^^,^^25^^,^^26^ but the transition from a predictive methodology to understanding gendered board access has not been explored.

To bridge this gap, we draw on BoardEx, the most commonly used data source for board composition and its connections, to collect data covering Canadian publicly traded firms from 2000 to 2022. This dataset provides affiliation trajectories for senior managers and directors, allowing us to map networks that change as careers evolve. It also provides information on their educational background and social interactions, which can be used to infer networks. We analyze these data by combining causal matching with sequence-based models, an approach that captures both career similarity and the dynamics of professional ties over time. This design enables us to move beyond static measures of representation and examine how career paths and network structures jointly shape access to boards.

By merging these perspectives, we aim to offer empirical evidence to inform debates on recruitment practices and diversity initiatives. Specifically, this paper seeks to examine the varied influence of different networks—educational, professional, and social—on board appointments, identify unique strategies used by male and female senior managers with analogous backgrounds to leverage these networks, and uncover systemic barriers that impede the advancement of women into board roles, proposing actionable insights to enhance gender diversity at the top of corporate governance.

## Methods

We begin by describing the data collection and curating process, then explain our matching methodology to make the results comparable, and finally describe the model we have designed to tackle the problem.

### Data

#### Professional network data collection

Our study uses an extensive dataset provided by BoardEx.^27^ The Canada Business Corporations Act^28^ requires all Canadian public companies to disclose information about their board members and senior management teams. BoardEx regularly updates this information. Following and extending the networking layers applied by Fracassi,^29^ we focused our investigation on the Canadian market, chosen for its diverse corporate structures and governance practices, offering a rich context for our analysis of networking dynamics.

The data collection and derivation process involved a systematic extraction of information from BoardEx, focusing on the top management teams and boards of over 700 publicly traded Canadian firms. For this study, a public Canadian firm is defined as an entity headquartered in Canada, listed on a Canadian stock exchange, and maintaining its primary communication address within Canada. The resulting dataset covers nearly 19,000 individuals during the 2000–2022 period. According to the company, “BoardEx data come from publicly available sources such as regulatory filings, company websites, annual reports, and press releases, collected since 1999, then verified and enriched by a large team of analysts using technology to map corporate and individual networks for detailed insights into boards, senior management, and their connections.” The data include sociodemographic information that, while complete, has some limitations, such as only having binary genders. For more information, we refer the reader to the BoardEx documentation.^30^

#### Network construction

The BoardEx database provided two distinct levels of data: board and individual. At the board level, BoardEx compiled comprehensive data on board members and top management groups of all publicly traded Canadian firms, including detailed employment histories. The tenure of a given individual in a particular position in a Canadian public company between year x and year y was documented. A role distinction was made between the board (either “executive director” or “supervisor/independent director”) and non-board (but still in the top management team). These data facilitate the construction of pairwise employment network connections. A connection is established between “current employment” (CE) when two individuals are employed concurrently in the same company, on the board, or in top management. Once an individual leaves the company, this connection transitions to a “prior employment” (PE) connection, reflecting the historical employment network connection.

At the individual level, BoardEx provided detailed information on each person’s educational background and social engagement trajectories. For the “education” (EDU) network, connections are established between individuals who attended the same university and graduated within a year of each other. For example, if one person graduated from a university in 1990 and another person graduated from the same university in 1991, they are connected within the EDU layer of the network. In addition, BoardEx cataloged the social engagement trajectories of individuals. From this information, we derived “social engagement” network connections. A “current social engagement” (CSE) connection is identified between individuals if they share memberships in clubs, organizations, or charities and have active roles in them. An active role was defined as one that included more than simply membership, except in the case of clubs. Common active roles included “trustee,” “president,” “advisor,” “board member,” and others. “Prior social engagement” (PSE) connections are established when an individual ceases to play an active role, thus representing their past social engagement within the network.

We note that not all connections captured in these layers represent direct interpersonal interactions. The ties between educational and social engagement can reflect implicit connections, grounded in shared institutional affiliation, similarity of experience, or overlapping social environments, rather than direct interaction. However, such affiliation-based ties can imply meaningful social capital. Recent research shows that professional, political, and educational connections between directors significantly shape corporate decision-making and risk-taking, underscoring the relevance of these affiliation-based connections in board contexts.^31^ Similarly, sociological evidence shows that elite hiring often operates through cultural matching processes, where common backgrounds and institutional affiliations function as signals of fit and trust.^32^

Data at the board and individual levels were used to create the following five professional networks.

• EDU: a connection in the EDU network is identified when two individuals graduate from the same university within 1 year of each other. One might argue that many Canadian universities have many students and that this condition might only be weak. However, strong bonds are formed during university years, and we included this network for this reason. Even with some noise, the network analysis will be able, as shown later in the paper, to capture which links are relevant.
• CE: a connection is established in the CE network between two individuals who concurrently serve the same company, either on its board of directors or on its senior management team.
• PE: a connection is established if two individuals work concurrently in the same company, either on the board of directors or within the top management group, and at least one of the individuals has left the company.
• CSE: people are considered socially connected if they share active roles in clubs, organizations, or charities.
• PSE: people are considered socially connected if they have previously shared active roles in clubs, organizations, or charities.

#### Quantification of network position

To fully quantify the importance of individuals within these networks, we calculated five centrality measures, each providing a unique insight into the position of individuals within the network.^33^^,^^34^ These five measures were as follows.

• Degree centrality: a measure of the number of direct connections that an individual has in the network. This measures the size of the individual’s immediate social network and thus how connected a person is to the network.
• Betweenness centrality: a quantification of the number of times an individual lies on the shortest path among all pairs of individuals in the network. High betweenness indicates that an individual often acts as a bridge in the network, connecting different communities within it. This measure highlights the role of an individual as an intermediary within a network.
• Closeness centrality: a calculation of the closeness of an individual to all other individuals in the network, based on the shortest paths, thus quantifying the individual’s ability to easily reach other individuals. This type of centrality indicates the ability to access and pass information through the network efficiently.
• PageRank centrality: derived from the algorithm used by Google^35^ to rank websites, this assesses the importance of an individual within the network based on the number and quality of their connections. This type of centrality indicates how well connected the individual is to other well-connected people in the network.
• Personalized PageRank (PPR) centrality: a variation of PageRank, specifically tailored in our study to emphasize connections with current board members of publicly traded Canadian firms. This metric assesses the importance of an individual relative to current board members and therefore the ability of board members to influence the importance of the individual in the network.

Following previous research on extracting influence from fraudsters and defaulters,^25^^,^^26^^,^^36^ we tailored the personal PageRank measure to highlight the significance of connections with current board members among target firms. Specifically, the target nodes under this measure for any given year *t* were identified as members of the boards of directors of all publicly traded Canadian companies in the previous year *t* −1. This approach allowed us to effectively assess the significance of connections with current board members in year *t* and quantitatively assess the influence of such connections on the likelihood that an individual is appointed to a board. The complete correlation matrix of these 25 centrality measures is reported in Table S1.

To further explore how professional networks impact director appointments, we modified the PPR algorithm to examine the influence of gender. We created two versions of the algorithm: one focused only on the impact of female directors and the other focused on male directors who were in the position from the previous year. This approach allowed us to study whether individuals tended to help others of the same gender more in professional networks and how this influenced corporate advancement. By comparing the influence of male and female directors separately, our aim was to uncover how gender had played a role in networking within corporations. This comparison would help us understand whether and how gender influences the support environment within professional networks, offering insights into promoting gender diversity in boardrooms. Our goal was to obtain a clearer view of how gender interactions within networks could shape the path to board appointments, providing guidance for developing strategies to encourage more balanced gender representation on the board.

**Figure 1:**
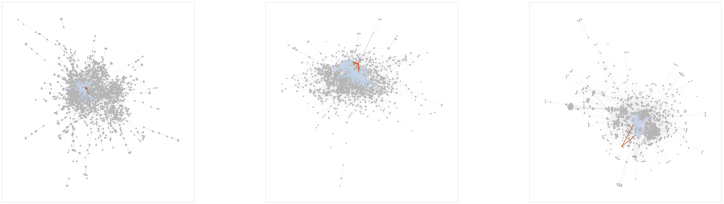
Year 2020 sample network visualization Example of a focal director’s connections in 2020 across current employment (left), education (middle), and social engagement (right) networks. First-degree ties (red) and second-degree ties (blue) are shown in distinct colors, with all other nodes muted. All three networks expand quickly from direct to extended ties, illustrating the richness of relational information. Their structures differ: the education network forms a concentrated core that quickly thins out, the employment network shows a more moderately clustered pattern, and the social engagement network is more evenly distributed across nodes without strong clustering. These contrasts illustrate how different tie types embed individuals in distinct positions within the broader corporate network.

In our study, we constructed a separate network for each source annually, resulting in five sequences of networks. For illustration, Figure 1 presents the networks of a focal director in 2020 across CE, EDU, and social engagement channels. Each image highlights the focal director and their first- and second-degree ties, showing how professional relationships extend through multiple layers of connections. For each network source, we computed the five centrality measures, which provided a quantitative assessment of the relative status and influence of an individual for our analyses.

Furthermore, to ensure comparability across different scales inherent in each centrality measure, we standardized the network centrality scores annually. Consequently, every individual in our dataset was assigned a total of 25 distinct scores annually, corresponding to the five network sources and five centrality measures per source. It is important to note that, due to variations in career lengths and trajectories, the lengths of time-series datasets for professional networks differed between individuals. Due to this variability, not everyone had the same duration of network data, a factor that we accounted for in our analytical approach by applying a recurrent neural network (RNN) with masking techniques. This also showcases how deep learning (DL) methods can deal with sequences of varying length, something that often challenges traditional methods.

The network scores for individuals were calculated using complete network data for any given year, ensuring an accurate reflection of each person’s network status within that year. However, for our final analysis, we retained a maximum of 10 years of network scores leading up to an individual’s initial board appointment. For example, if an individual’s appointment occurred in 2013, we included network scores for that individual from 2003 to 2012. For those who were never appointed to a director role, we incorporated up to 10 years of network scores until the end of their career documentation. We refer to this time as the active period of an individual. This focus on the most relevant period for board appointments helped maintain the integrity of our predictive analysis.

Furthermore, it is important to note that if an individual’s data extended only until a year (e.g., 2005) before the end of our initial training period (years 2000–2009), their data were excluded after the network scores were computed. This approach aligned with our goal of predicting first-time board appointments after 2010 and maintained the integrity of predictive analysis by focusing on those individuals for whom a complete and relevant data history was available.

### Summary statistics

Following our detailed definition and construction of the networks, our refined dataset covered 19,395 individuals, with more than two million connections, in 772 publicly traded Canadian companies, from 2000 to 2022. We focused on leveraging these networks to predict the likelihood that an individual would be appointed as a board member for the first time in any of the 772 companies in the following year.

We obtained three types of data for each individual: demographic characteristics (gender, educational qualifications, nationality, and professional experience), career features (job title, sector, organization, and index of positions held), and the network centrality measures.

#### Demographic and career composition

Table 1 presents the number of senior managers in each year from 2010 to 2021 for the companies in our dataset. The table also identifies how many and what fraction of those senior managers advanced in position to their first director role in the following year. In this way, the table highlights the changing nature of our dataset.

**Table 1:** Annual dynamics of (first-time) director appointments

Year	Total senior managers	Directors appointed in the next year (total)	Directors appointed in the next year (male)	Directors appointed in the next year (female)	Fraction % (male)	Fraction % (female)
2010	6,764	327	288	39	88.07	11.93
2011	7,338	275	245	30	89.09	10.91
2012	14,627	291	249	42	85.57	14.43
2013	14,474	290	201	89	69.31	30.69
2014	14,187	235	180	55	76.60	23.40
2015	13,797	253	196	57	77.47	22.53
2016	13,280	256	182	74	71.09	28.91
2017	12,679	242	166	76	68.60	31.40
2018	11,977	213	143	70	67.14	32.86
2019	11,271	232	150	82	64.66	35.34
2020	10,491	239	147	92	61.51	38.49
2021	9,761	195	109	86	55.90	44.10

Detailed year-by-year statistics regarding the number and fraction of first-time male and female directors appointed across publicly traded Canadian companies from 2010 to 2021. This table demonstrates the evolving gender dynamics in corporate board appointments, reflecting broader trends toward gender diversity.

Table 2 presents the distribution of the active periods of the executives.

**Table 2:** Distribution of active years for individuals

Years	10	9	8	7	6	1–5
%	73.29	2.88	2.78	3.04	3.13	14.87

Distribution of the lengths of the executives’ active periods, highlighting the years of active network engagement.

**Figure 2:**
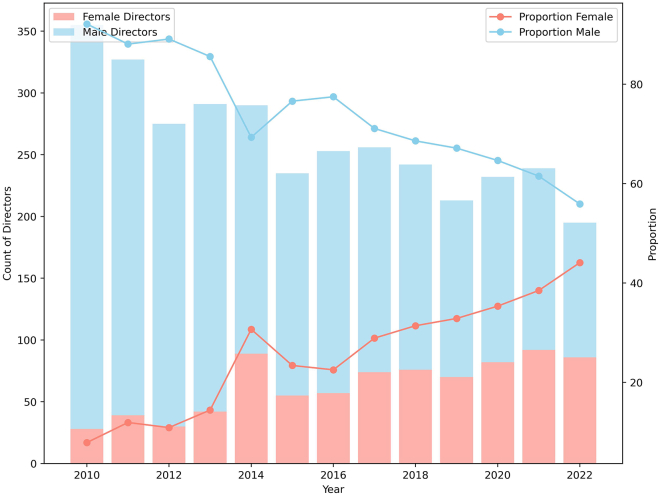
Number of director appointments across time by gender Per-gender trends in board appointments, illustrating the evolving representation of men and women over time and highlighting recent progress toward greater gender diversity in corporate boards.

Our dataset comprised 15,167 (78%) men and 4,228 (22%) women. Among these, 17% of men and 19.4% of women eventually secured board positions. The trend in first-time board appointments, shown in Table 1 and Figure 2, signifies a gradual increase in gender diversity. This increase reflects the impact of initiatives aimed at improving boardroom gender balance beginning in 2015, when the Ontario Securities Commission recommended that companies comply or explain disclosure regarding governance,^37^ albeit without a legal mandate to promote gender diversity on boards in Canada.

This demographic overview, coupled with the evolving pattern of board appointments, demonstrates changes in board composition that are the potential result of ongoing efforts to address gender disparities within the realm of corporate governance, resonating with broader academic and policy discourse aimed at promoting diversity at the highest levels of corporate leadership.

#### Network scores by gender

We conducted a detailed analysis of network scores and centrality measures, revealing possible gender-based differences in director appointments within public Canadian corporations. We examined various networks as previously described, each providing information on how gender influences networking dynamics. Selected examples have been highlighted in the main text to illustrate these differences.

**Figure 3:**
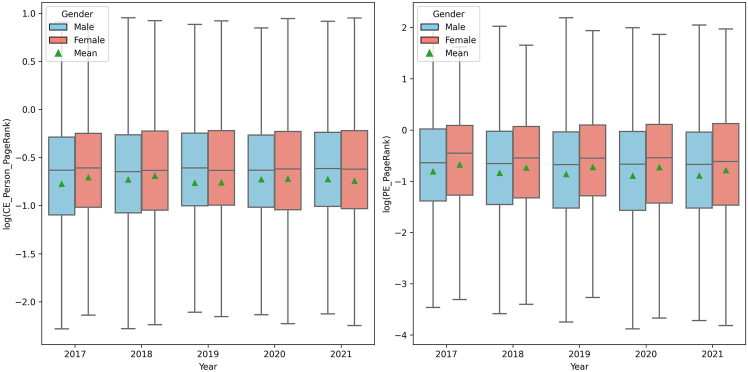
Boxplot of working networks over time by gender Comparison of the logarithmic values of standardized personalized PageRank centrality scores within current employment networks (left) and PageRank centrality scores within prior employment networks (right), by gender, from 2017 to 2021.

In professional networks (Figure 3), CE networks evaluated using PPR showed little variability in median scores over time for both genders. Men exhibited a slightly broader range of scores in earlier years, which became comparable to the range for women in later years. Similarly, in previous employment networks, assessed through PageRank, the median and mean scores for both genders remained stable, with female executives generally achieving higher median and mean scores compared to male executives. The interquartile range for men showed more variability, suggesting a broader spectrum of historical network centrality among male executives. These observations imply that, while gender-based disparities existed in the range of influence within professional networks, the central tendency of influence remained similar between genders in both CE and PE contexts.

**Figure 4:**
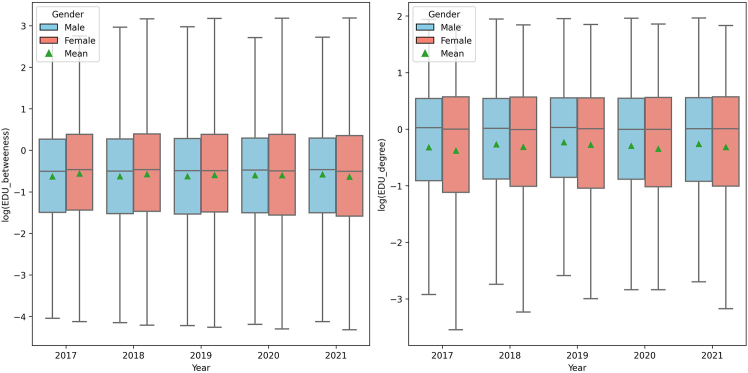
Boxplot of education networks over time by gender Comparison of the logarithmic values of betweenness centrality (left) and degree centrality (right) scores within education networks, by gender, from 2017 to 2021.

Analysis of the EDU network (Figure 4) using betweenness and degree centrality metrics revealed notable patterns by gender. The betweenness centrality scores were relatively stable over time for both genders, with women slightly higher on average, suggesting their crucial roles as key connectors within EDU networks. For degree centrality, both genders maintained stable mean and median values, but female executives displayed a broader interquartile range and a wider overall score range, indicating greater variability and more extensive networking engagement among female executives.

**Figure 5:**
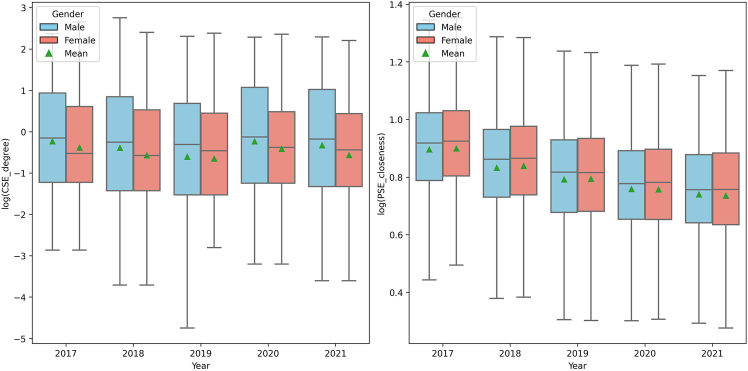
Boxplot of social engagement networks over time by gender Comparison of the logarithmic values of degree centrality scores within current social engagement networks (left) and closeness centrality scores within prior social engagement networks (right), by gender, from 2017 to 2021.

Regarding social connections, our examination of current and past social connections (Figure 5) highlighted the distinct gender differences in networking behaviors. For CSE, explored through degree centrality, there is an apparent difference between men and women over the 5-year period. Whereas women consistently have a lower median degree centrality, men have a larger interquartile range, indicating greater variability in their social connections. In PSE, assessed through closeness centrality, both genders showed a clear pattern of declining median and mean centrality scores over time, with this trend being somewhat more pronounced in women, suggesting a faster decrease in social capital in past engagements. These trends underscore gender-specific strategies in the formation and maintenance of social networks, critical for understanding the impact of networking on career development.

Our findings revealed gender-specific differences between professional, educational, and social networks, which further justify our subsequent analysis. In professional networks, the broader range of centrality of male executives indicates a diverse influence, while female executives established a notable presence in EDU networks as central connectors, a strategic positioning likely to enhance career advancement opportunities. In social networks, despite a lower median centrality in current engagements, women maintained relevance in past roles, indicated by a less significant decrease in centrality. These insights into network scores and gender centrality measures underscore the importance of professional networks in shaping corporate board trajectories and illuminate complex gender influences on these dynamics, highlighting the need for a more in-depth understanding of gender-specific networking strategies within corporate content, as described in the next section.

### Matching methodology for causality analysis

A key challenge in assessing the role of networks in board appointments is distinguishing correlation from causation. Individuals with different career trajectories may also differ in their network positions, making it difficult to determine whether the networks themselves exert an effect or simply reflect prior career advantages. Although the problem of DL-based causality is still a source of active research, we can begin to address this issue by adopting a matching design that pairs women with men who have highly similar career sequences and demographic profiles. This design provided a closer approximation of a counterfactual comparison: what would the board membership outcome of a woman look like had she followed the same career path as a comparable man?

The procedure was carried out in four steps. First, we encoded directors’ career histories as sequences of standardized job categories, sectors, and organizational types. Second, we calculated a dissimilarity matrix using these sequences to quantify differences in career paths. Third, we incorporated demographic covariates, including age and education, to strengthen comparability. Finally, we implemented gender-based matching, pairing each woman with the closest male counterpart according to the combined dissimilarity score. By keeping both career trajectories and demographic features constant, this approach allowed us to isolate the additional contribution of networks to board appointments. The following steps operationalized this design by systematically encoding and comparing career sequences to enable robust gender-based matching.

#### Sequence construction

Transforming career attributes into sequence data was a critical step in our matching process, allowing us to accommodate the complexity and variability of professional trajectories effectively. To manage the breadth of career histories, with some spanning more than 100 positions, we focused on the 15 most recent roles, a range that 96% of individuals did not exceed, aligning our analysis with the most relevant stages of career development. This limitation is essential to maintain the manageability of the data and to ensure a focus on the critical phases that lead to board appointments.

Each role within these sequences was categorized into four dimensions, namely job title, sector, organization type, and index, resulting in four separate sequences for each individual. Consequently, four distinct sequences were generated for each individual, providing a multi-perspective approach to measuring career trajectories. The construction of a dissimilarity matrix for each dimension, combined with demographic characteristics, formed the basis for our comprehensive dissimilarity analysis. The details of the four dimensions are as follows.

• Job path: the job titles were categorized into 18 different types to reduce variability and improve interpretability. Detailed categories can be found in Note S1.
• Sector path: the sectors in which the individuals worked were classified into 53 distinct sectors, as defined by BoardEx. This categorization allowed for the capture of sectoral shifts or consistencies, providing insight into sector-specific career dynamics.
• Organization-type path: BoardEx categorized the types of organizations into three distinct categories: public companies, private companies, and others (such as government or clubs).
• Index path: comprising 18 different indexes, as defined by BoardEx, the index path provided additional indicators of career progression and organizational character. Detailed categories can be found in Note S2.

For sequence data analysis, we used the transition rate matrix (TRATE^38^) method, calculating substitution costs based on observed transition rates between different states. This method, documented for its efficacy in capturing the subtleties of categorical data sequences, operates on the principle that more frequently observed state transitions carry lower transition costs. For example, a transition from “vice president” to “president,” if observed more commonly than a transition from “manager” to “C-suite,” would incur a lower transition cost. Furthermore, the order of transitions was also considered; a common transition from “vice president” to “president” would have a lower cost compared to the less frequent reverse transition from “president” to “vice president.”

The TRATE method enabled the creation of a matrix that mirrored career transitions within our dataset, grounding our dissimilarity calculations in real-world career patterns.

#### Dissimilarity matrix with sequence data

After converting career attributes into structured sequences and using the TRATE method to calculate transition costs, the next step was to create dissimilarity matrices for each of the four career dimensions. These matrices quantitatively measured the differences in the career trajectories of individuals, capturing the unique aspects of each person’s professional journey.

We used the optimal matching (OM) technique to calculate the differences between sequences. The OM method is well known for its ability to quantify the dissimilarity between sequences, and it has been extensively used in social science to study career sequences.^39^^,^^40^^,^^41^

The OM process focused on identifying the minimum number of operations, that is, insertions, deletions, or substitutions, required to transform one sequence into another and the cost of these operations derived from the previously calculated transition costs. The more operations and higher costs required for this transformation, the greater the observed dissimilarity between the two sequences. The OM distance *d*(*i*,*j*) between the sequences *S*_*i*_ and *S*_*j*_ can be expressed as

$$d\left(i,j\right)=\min\left(\sum_{k=1}^{n}c_{k}\cdot \delta_{k}\right)\text{,}$$

where *c*_*k*_ represents the cost of the *k*-th operation (insertion, deletion, or substitution), calculated from the previous step; *δ*_*k*_ is an indicator function that takes the value of 1 if the *k*-th operation is performed and 0 otherwise; and *n* is the total number of operations needed to transform sequence *S*_*i*_ into sequence *S*_*j*_.

This approach yielded a 19,395 × 19,395 dissimilarity matrix for each dimension of the career path. For further detailed mathematical expressions and the full algorithm, please refer to Method S1.

To ensure comparability across these matrices, we normalized each dissimilarity score, setting the lowest value to 0 and the highest to 1. A dissimilarity score of 0 would indicate identical paths, implying that no transformation costs or operations were required, a scenario typically observed in self-comparisons. In contrast, the maximum value of 1 would represent the most dissimilar pair, indicating the highest costs required for sequence transformation.

These matrices provided a rich, detailed view of the varied and complex career paths within our dataset. They formed an integral part of our analysis and laid the groundwork for pairing individuals based on their career trajectories.

#### Integration of static demographic features

Together with dynamic sequence data, our analysis also incorporated static demographic characteristics of executives to provide a comprehensive assessment of each individual’s history. This integration included educational qualifications, nationality, and professional experience, each uniquely contributing to understanding career development patterns.

• Educational qualifications: the inclusion of educational qualifications, such as Master of Business Administration (MBA), Juris Doctor (JD), and Doctor of Philosophy (PhD), was based on recognition of their significant role in career development. These qualifications often serve as gateways to higher-level positions and are associated with enhanced skills and knowledge, which are crucial to fulfilling leadership roles.^42^^,^^43^ The Hamming distance metric^44^ was used to calculate differences in educational background between individuals. This approach provided a quantifiable measure of how educational qualifications varied across the dataset, and the resulting matrix was normalized to align with other components of our analysis.
• Nationality: nationality provides information on the diverse cultural and geographical demographics of professionals. Recognizing the wide variety of nationalities, we grouped all nationalities appearing in the dataset into 12 distinct categories, listed in Note S3, to manage the variability. The nationality data were transformed using one-hot encoding to convert categorical values into a binary matrix. The dissimilarity in nationalities was then calculated using the Hamming distance metric, which is suitable for binary data. The resulting dissimilarity matrix was normalized to ensure uniformity in scale.
• Professional experience: the difference in professional experience, measured in years, was calculated using the Euclidean distance metric. This metric is well suited for continuous data and helped quantify the degree of variance in professional experience among individuals. The normalization of this matrix allowed for consistent comparisons with the other dimensions of the dataset to be performed.

The culmination of our analytical process was the construction of a comprehensive combined dissimilarity matrix. This matrix represented an integration of normalized dissimilarities derived from both the dynamic aspects of career sequences and static demographic features. The resulting matrix was a 19,395 × 19,395 construct; each entry in the matrix spans a range of values from 0 to 7, and each value corresponds to the aggregate dissimilarity between the seven distinct matrices. This integrated matrix formed the basis for our matching methodology and subsequent analysis.

#### Implementing gender-based matching using the dissimilarity matrix

The comprehensive dissimilarity matrix, which encompasses both career sequences and static demographic characteristics, underwent an additional normalization process. This refinement was essential to scale the values within a range from 0 to 1, where 0 represented identical career trajectories and demographic characteristics (typically observed in self-comparisons) and 1 signified the least similar individual in the dataset.

In our study, we emphasize gender-based matching by using a dissimilarity threshold of 0.15 to pair candidates with similar demographics and career paths. For effective matching, we selected male counterparts for female candidates based on the closest dissimilarity score, which was also required to be smaller than the threshold, ensuring a one-to-one match without repeating assignments. This strategy resulted in our successful match of approximately 97% of the 4,228 female candidates. This high matching ratio showcased a notable early result: for nearly every female director in the dataset, it was possible to find a similarly skilled male director; therefore, the choice of one versus the other had been situational. This result is contrary to those of most propensity matching exercises, in which significant amounts of data were dropped to achieve the matching.^45^ In our case, having a high match ratio indicates that the results of this work are applicable to the entire set of female directors from our data.

**Figure 6:**
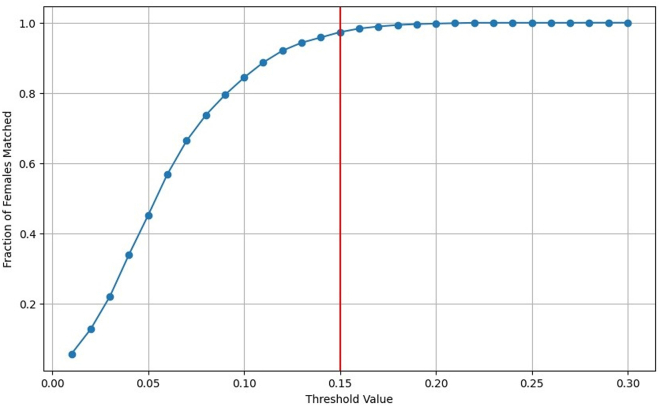
Fraction of women matched This figure demonstrates the relationship between various dissimilarity score thresholds and the percentage of female candidates matched with male counterparts. The graph highlights our strategic threshold choice to optimize both match quality and coverage.

Figure 6 visualizes how the chosen threshold influenced the proportion of women matched without repeating male assignments. To achieve matches for all female candidates, a minimum dissimilarity score of 0.28 would have been required; however, our selection of 0.15, while more stringent, successfully matched 97% of women.

Post-matching appointment rates further validated our matching process, showing comparable rates for women (18.4%) and men (17.6%), with a chi-squared test that confirmed no significant differences between the two genders among the matched samples.

Following this matching process, we completed our matched dataset. In the following subsection, we describe the models and methodology applied for performance assessment in our study.

### Model and assessment

#### Model description

Our study integrated the long short-term memory (LSTM) model to analyze the sequential nature of professional network data, addressing the challenge of capturing the temporal dynamics of network connections and their impact on board director appointments. LSTM, as introduced by Hochreiter and Schmidhuber,^46^ was designed to overcome the vanishing gradient issue common in traditional recurrent neural networks, making it effective for modeling complex and evolving sequences, such as node centralities extracted from networks. This architecture is particularly well suited to our setting, as directors’ careers unfold as ordered sequences in which both the timing and progression of the roles are informative. By modeling these temporal dynamics, the LSTM enables us to evaluate how career paths and network positions jointly shape the likelihood of board appointments.

**Figure 7:**
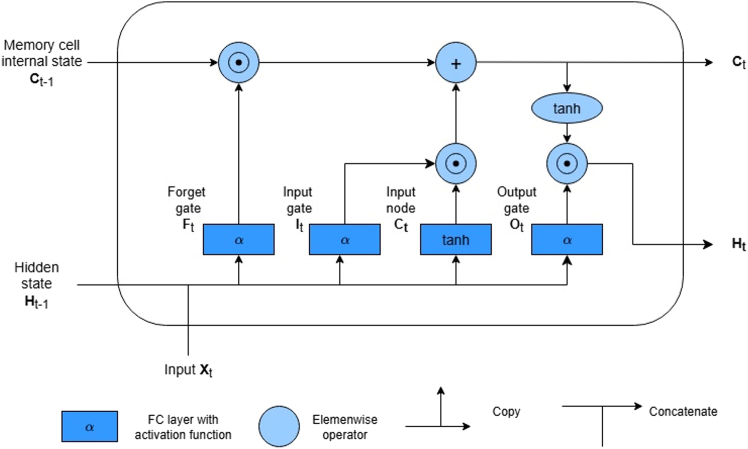
Schematic of an LSTM unit architecture This graph demonstrates the schematic of the LSTM unit architecture, illustrating the interplay of input, forget, and output gates essential for processing sequential data.^47^

The mechanism of LSTM includes a series of input, forget, and output gates, as shown in Figure 7. These gates allow such a model to selectively remember and forget information, a feature that was essential for modeling the nonlinear and evolving nature of professional networks and career trajectories. The ability of LSTM to process sequences of varying lengths was particularly advantageous for our dataset, in which individuals possessed different network spans and data availability.

For our analysis, the LSTM model processed a multivariate time series composed of 25 data points per individual per year, encompassing all network sources and centrality measures. In focused analyses of specific aspects of the network, such as CE, the model processed a reduced set of five annual data points. This granularity allowed for a detailed examination of how particular aspects of the network had influenced the likelihood of attaining board positions. We used a dropout rate of 0.5, as introduced by Zaremba et al.,^48^ to mitigate the risk of overfitting, a common challenge in deep learning models. Following the LSTM layer, a fully connected linear layer mapped the hidden state to a single output value per sequence, indicating the projected probability of a board appointment in the following year.

To accommodate the varying sequence lengths in our dataset, we employed a front-padding technique, aligned with best practices in neural network training for variable-length sequences.^49^ This method involves padding shorter sequences with zeros at the beginning to ensure uniform input lengths for the LSTM model. During the forward pass of the model, a masking step was applied to the output of the LSTM layer, focusing the model’s attention only on the meaningful and unpadded parts of the sequence.

Our choice of binary cross-entropy with logits (shown in Equation 1) as a loss function aligned with the binary nature of our target variable (director appointment: yes or no). This decision was in line with standard practice for binary classification tasks in neural networks.

(Equation 1) $$L=-\frac{1}{N}\sum_{i=1}^{N}\left[y_{i}\;\log\left(p_{i}\right)+\left(1-y_{i}\right)\log\left(1-p_{i}\right)\right]\text{,}$$

where *N* is the batch size, *y*_*i*_ is the true label (0 or 1) for the sample *i*^*th*^, and *p*_*i*_ is the predicted probability that the sample *i*^*th*^ belongs to the positive class, representing the true label equal to 1.

The hyperparameters of the model, including the learning rate, dimensions of the hidden layer, and training epochs, were optimized using a grid search methodology to ensure optimal model performance. We used an Adam optimizer based on its efficiency in addressing sparse gradients and its adaptability to different contexts.^50^ Given the number of models in this study, we have presented the hyperparameter setup and optimized parameters for all models in Note S4 and Table S2, respectively.

#### Performance assessment

We adopted the out-of-time cross-validation single-step approach (OOTCV-SSA) algorithm^51^ to train and validate the LSTM model; this technique underscored the adaptability and accuracy of the model over time in evolving professional networks. First, we used data from 2000 to 2009 for training, offering a firm historical foundation. The testing then incrementally added data from subsequent years, starting with data from 2010, adopting a method in which each year’s test data expanded the training dataset incrementally. The OOTCV-SSA algorithm allowed continuous model refinement and assessment, with the first testing phase being performed using 2010 data and subsequent testing being performed on data from the following years, expanding the training dataset annually. This procedure not only facilitated an evaluation of the predictive performance of the model over time but also provided information on its evolving efficacy as newer data were incorporated. This design is particularly effective in our context, as it mirrors the temporal unfolding of directors’ careers and networks, ensuring that predictive performance is assessed in a forward-looking manner rather than with shuffled samples. This provides a stronger and more realistic assessment of how career and network dynamics influence board appointments over time.

To analyze the performance of the model with a focus on recent results, we implemented an exponentially weighted area under the receiver operating characteristic curve (AUROC) method, also known as the area under the curve (AUC) method, given its widespread use. This technique prioritized the performance of the model in recent years by assigning greater weights to the results in the last few years, reflecting the dynamic nature of professional networking. Through this method, the AUC scores for each year were weighted, with more recent years given greater emphasis. The outcome, an exponentially weighted average of AUC scores, provided an aggregate measure of the performance of the model.

Various segments of the dataset, covering different sources and measures of the network, were individually subjected to training and refinement within their respective LSTM models. This segregated approach allowed for an in-depth exploration of the predictive capacity of each network segment regarding board director appointments. For example, models trained solely on data from specific sources, such as CE, addressed the unique impact such network aspects have on securing board positions. The fine-tuning phase for each model variant involved a customized grid search for optimal hyperparameters, including the learning rate, hidden dimensions, and the number of training epochs, specific to each data subset. The resulting AUC scores from these specialized models offered a nuanced view, revealing the multifaceted factors that influence the probability of achieving board director appointments.

#### Assessment of feature importance over multivariate analysis

In our effort to investigate the impact of individual network sources on board appointments, we conducted a feature ablation study,^52^^,^^53^ a method that is well suited to evaluate our LSTM model that processes complex temporal data structures. This study aimed to investigate and explain the significance of each network source and centrality measure over time, providing clear insights into their contributions.

First, we trained an LSTM model with optimal parameters derived from the full scope of network data, establishing a performance benchmark for the original model in our time-series dataset. This benchmark facilitated subsequent comparisons with modified models from which specific network sources had been systematically masked. By setting the values of selected network sources to zero, we isolated their effects, maintaining the integrity of the remaining data. This replacement with zero is not meant to represent a literal absence of ties but rather functions as a neutral mask, following standard ablation practice. It suppresses the informational signal of the targeted features while keeping the input structure unchanged, allowing performance differences to be attributed specifically to those features. Two approaches can be used to validate the contribution of network-based inputs: comparing against a null model (e.g., shuffled networks) or applying feature ablation. In line with common practice in ML and explainable AI,^54^^,^^55^^,^^56^ we adopt the ablation approach to quantify the contributions of marginal features within our predictive pipeline.

Second, the influence of each source of masked network on the performance of the model was assessed by observing changes in the weighted area under the curve (AUC). The network source causing the most significant decrease in weighted AUC after removal was identified as the most influential. The degree of influence of each feature was normalized to quantify the significance of the feature. Similarly, we evaluated the importance of centrality measures individually, excluding each from the LSTM input data, thus determining their distinct contributions to the predictive capability of the model.

This methodical feature ablation process, consistently applied over successive years, provided a longitudinal view of the evolving significance of each network source and centrality measure. These temporal insights allowed for a nuanced understanding of the changing dynamics affecting board member appointment within professional networks.

The adoption of this ablation strategy allowed us to navigate the limitations associated with direct interpretation techniques such as Shapley additive explanations (SHAP),^57^ which might have struggled with sequential data of varying lengths. Therefore, this design was well suited to the objective of our study of identifying and quantifying key network sources and centrality measures that drive board director appointments. Crucially, the ablation approach also enables us to evaluate whether network-based variables add explanatory value beyond career trajectories and demographics. By systematically masking sources and centralities, we isolate the distinct contribution of networks to board appointments, directly addressing our research aim of understanding how professional connections shape gendered pathways into leadership.

#### Analysis of retain-forget behavior

To further examine the internal dynamics of the LSTM, we analyzed the retain-forget behavior of the recurrent gates. This analysis complements the feature ablation from the previous section by highlighting when and how different network features are preserved or discarded throughout the directors’ career histories. In recurrent networks, the forget and input gates regulate how much of the past state is preserved and how much new information is incorporated.^58^^,^^59^ This mechanism directly determines the effective memory of the model, and its analysis provides insight into which parts of the directors’ network histories are retained or discarded.

At each time step *t*, the LSTM computes a forget gate *f*_*t*_ and an input gate *i*_*t*_, which determine the extent to which past cell states are retained and new information is incorporated:

$$c_{t}=f_{t}⊙c_{t-1}+i_{t}⊙g_{t}\text{,}$$

where *c*_*t*_ is the state of the memory cell and *g*_*t*_ is the candidate update. To quantify gate dynamics at the group level, we define retention-intake indicators (RIIs) that combine gate activations with the magnitude of the input features of the corresponding group. Specifically, for a feature group *g* (e.g., one of the five network sources or one of the five centrality measures) at lag l, we compute

$$RII_{g,l}^{\left(f\right)}=E\left[f_{l}\cdot ∥x_{l,g}∥\right],RII_{g,l}^{\left(i\right)}=E\left[i_{l}\cdot ∥x_{l,g}∥\right]\text{,}$$

where $∥x_{l,g}∥$ denotes the $l_{p}$-norm of inputs from group *g* at lag l, and the expectation is taken across all directors in the validation fold. These indices reflect both the gating decision and the strength of the group’s input signal, providing an interpretable measure of temporal retention and intake. Related interpretability approaches for RNNs have similar combined gate activations or saliency with input magnitudes to attribute model behavior to features.^60^

This analysis allows us to visualize which groups of features are primarily remembered in the most recent years and which are discarded, thus clarifying the temporal focus of the model. Importantly, retain-forget dynamics capture the temporal memory of LSTM rather than the direct predictive contribution.^61^ Groups with high raw retention, that is, where more variables are retained in the LSTM flow, signaling that the LSTM has not filtered much of the data, including, potentially, noise, may contribute little to the accuracy of the out of sample, while others with modest retention may still be decisive for prediction, as the filters in the LSTM may have more strongly reduced noisy inputs to keep informative signals, consistent with prior findings on saliency and gate-based interpretability.^60^ This distinction complements the ablation analysis by showing that the model not only emphasizes certain sources and centrality measures but also prioritizes the recency of directors’ networks. In doing so, the retain-forget analysis strengthens our interpretation that board appointment outcomes are shaped most strongly by the most recent connections, although longer histories also inform the hidden state of the model.

In summary, our methodological approach begins with the construction of five professional networks of Canadian directors, capturing employment, EDU, and social engagement ties. From these networks, we derive centrality measures that quantify individuals’ positions within each layer. We then combine these measures with detailed career sequences and demographic characteristics to create a matched dataset. This matching process allows us to control for career path similarities while isolating the role of networks in shaping board appointments.

Building on this dataset, we apply an LSTM model to evaluate how directors’ trajectories and network positions jointly influence access to corporate boards. Model performance is assessed using out-of-time validation, and we perform a series of feature ablation exercises to identify the specific contribution of different network-based variables. We further analyze the retain-forget behavior of the LSTM to uncover how the model prioritizes recent versus historical information, thereby clarifying the temporal mechanisms through which network ties exert influence. Finally, by comparing the results for men and women, we highlight how gender conditions the pathways to Canadian corporate boards, linking our methodological design directly to the study’s research questions.

## Results

In our empirical examination, we navigated the complex dynamics of professional networks and their impact on gender disparities in board appointments. Based on our carefully matched sample, our objective was to obtain a causal understanding of how networks directly influence the likelihood of board appointment. Although the findings of our unmatched sample were informative, our focus remained on the matched sample to gain a deeper understanding of how gender influences professional networking within corporate governance. The exploration presented here sought to elucidate the pathways to board appointments for candidates with similar demographics and career trajectories, contributing to the broader dialogue on achieving equitable representation in the corporate sphere.

To assess whether these observed gender differences are statistically significant, we performed formal significance tests using z-tests derived from the reported 95% confidence intervals. The methodology and complete results for all figures in this section are reported in Method S2 and Tables S3–S8, respectively.

### Influence of network sources

#### Model results across different network sources

**Figure 8:**
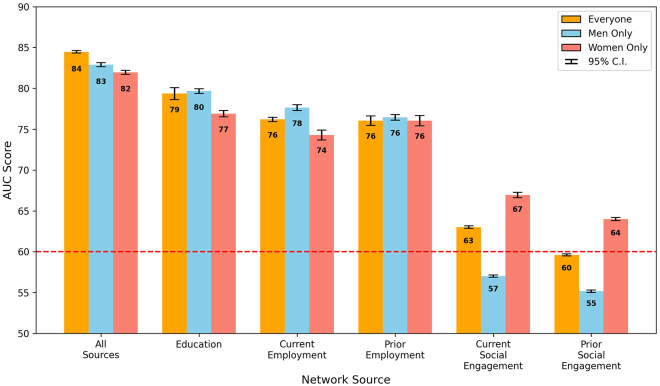
Network source AUC by group Model performance, measured in weighted AUC, across different professional network sources. This graph comparatively illustrates the predictive power (with a 95% confidence interval) of education, employment, and social engagement networks in determining board director appointments, with a focus on gender disparities. The dashed line at AUC = 0.60 represents the minimum threshold for a single network source’s influence to be considered significant.

The efficacy of various professional networks in influencing board director appointments is presented in Figure 8, in which each bar represents the outcome, measured in AUC, of a fine-tuned model with a 95% confidence interval. These models were optimized to analyze the impact of network sources on board member appointment, applied to the matched pairs of candidates, with the data segmented into three distinct groups for a comprehensive understanding based on a combined analysis including all candidates (“everyone”) and separate analyses focusing exclusively on male (“men only”) and female (“women only”) candidates. The “all sources” bar presented aggregated results from models that incorporated all five network sources as inputs, offering a comprehensive view of networking influence. In contrast, the remaining bars individually highlighted the model outcomes when solely one network source was used, pinpointing each source’s unique influence.

When accounting for similar professional histories, the overall influence of all network sources combined yielded an AUC score of 0.84 (shown as 84 in the figure) for everyone, with scores of 0.83 and 0.82 for men and women only (*p* <0.001), respectively. This result demonstrates a balanced influence at this collective level. It also suggests that when demographics and career paths are aligned, professional networks provide approximately equal advantages to both men and women in board director appointments.

Exploring specific network sources, EDU networks were particularly notable, with a minor differential: the women’s AUC at 0.77 slightly trailed the men’s AUC at 0.80 (*p* <0.001), suggesting a marginally greater utility of educational connections for men within matched contexts. Those familiar with the sheer size of many Canadian universities may find this network effect a bit surprising. However, several explanations are possible for this result, including that many directors studied in relatively smaller business or law faculties or may have been active in student government. Despite the small difference, both scores were high, indicating that EDU networks were important for the career advancement of both genders.

The examination of CE networks within the matched sample unveiled a gender gap: the AUC for men at 0.78 surpassed women’s at 0.74, indicating a relative advantage for men with current workplace connections (*p* <0.001). In contrast, PE connections exhibited equal AUCs of 0.76 for both genders, suggesting a gender-neutral lasting benefit from historical professional ties.

In social engagement networks, women’s adeptness at capitalizing on current social networks was reflected in a higher AUC of 0.67, compared to 0.57 for men (*p* <0.001). This effect underscores the pivotal impact of active social roles for women. Similarly, in previous social engagements, the women’s AUC at 0.66 significantly outperformed the men’s AUC at 0.55 (*p* <0.001), a number just above random chance, further advocating the strategic importance of social engagement in women’s professional advancement.

#### Temporal retention of network source

Although the preceding analysis identifies which network sources improve predictive performance, it does not indicate how long those signals remain influential within directors’ histories. We therefore apply the retention index described in the methods, which combines gate activations with source magnitudes to quantify how strongly information is carried forward across career stages. Higher values indicate that information from a source is preserved more strongly in the model’s memory.

Among paired candidates (Table S9), the average retention ranking is stable: EDU (2.28) and CE (2.19) show the highest mean values, followed by PE (1.90), and both forms of social involvement are lower (prior: 1.10; current: 0.89). Retention is also front-loaded across sources, with about 53%–59% of the remembered mass concentrated in the first five lags.

**Figure 9:**
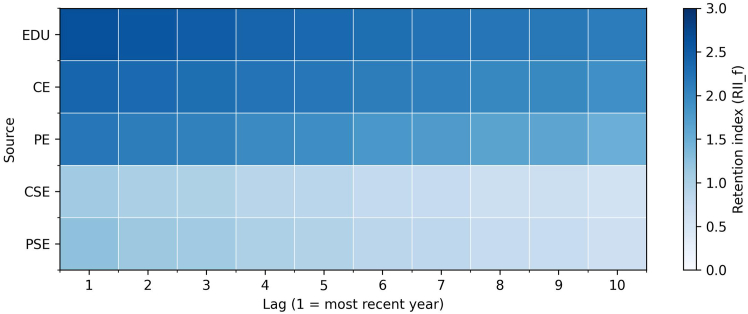
Retention analysis network sources for men Heatmap of the retention index across 10 lags for 5 network sources for men. Retention is concentrated in the most recent part of directors’ histories, with education and current employment preserved most strongly, followed by prior employment. Both forms of social engagement are retained less, and their signals fade more quickly in men’s trajectories.

**Figure 10:**
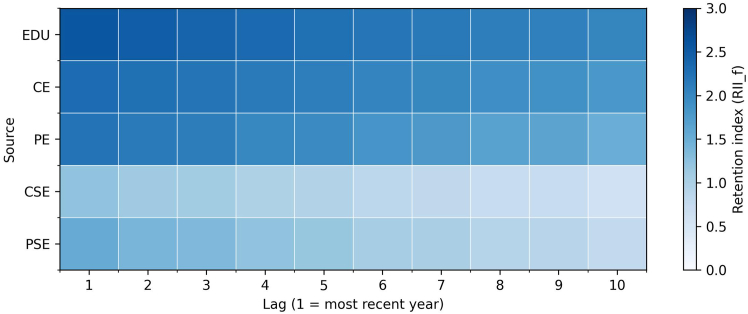
Retention analysis network sources for women Heatmap of the retention index across 10 lags for 5 network sources for women. Retention is concentrated in the most recent part of directors’ histories, with education and current employment preserved most strongly, followed by prior employment. Both forms of social engagement are retained less, but their signals persist more visibly in women’s trajectories.

Figures 9 and 10 display gender-specific patterns. For men, retention is concentrated in the most recent career stages, with EDU (2.34) and CE (2.14) preserved most strongly; PE (1.85) is next, while PSE (0.92) and CSE (0.82) are weaker and fade rapidly. For women, EDU (2.25) and CE (2.06) also dominate, but PE (1.88) is slightly higher than for men. More importantly, both forms of social engagement are retained more strongly in women’s trajectories: PSE (1.13 versus 0.92 for men) and CSE (0.90 versus 0.82 for men). The same front-loaded pattern holds but with greater persistence of informal activities for women.

Together, these results show that while EDU and employment dominate the memory of the model for all directors, women’s pathways preserve more from social involvement alongside formal career signals. This highlights that informal activities contribute more consistently to women’s trajectories, even when formal credentials are present.

#### Breakdown of network source contribution

**Figure 11:**
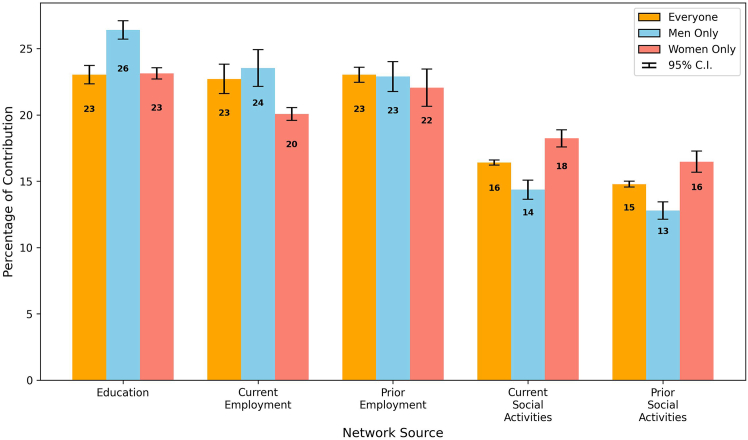
Network source contribution breakdown A bar plot showing the comprehensive breakdown of the weighted contribution of different network sources to the likelihood of board appointments, including a 95% confidence interval. The plot reveals the comparative significance of various networks in shaping boardroom paths.

**Figure 12:**
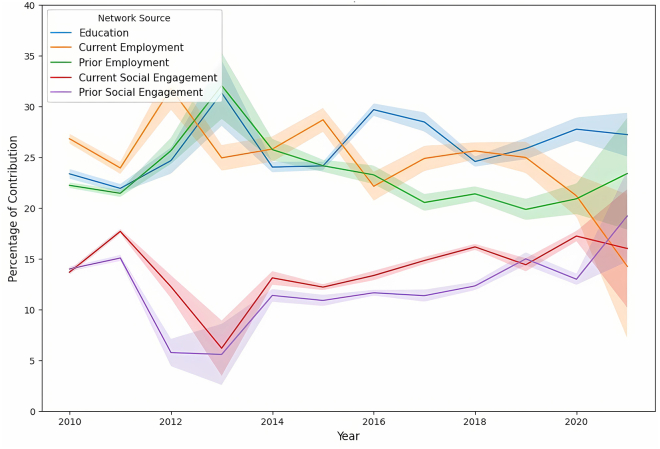
Time-series analysis of the significance of different network sources for men Time-series analysis of the significance of different network sources for male candidates. The shaded area represents the 95% bootstrap CI for the trends.

**Figure 13:**
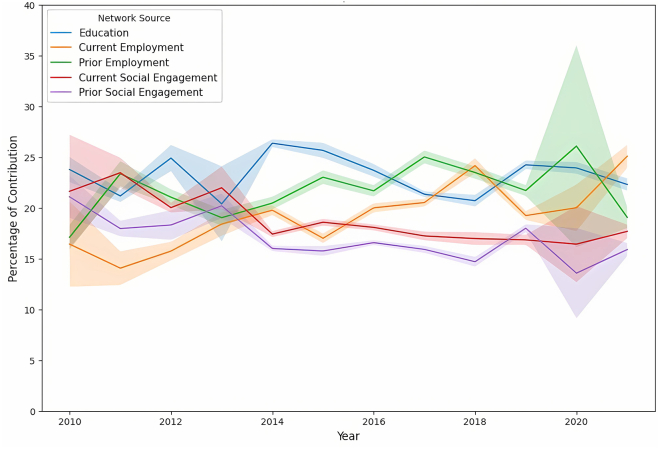
Time-series analysis of the significance of different network sources for women Time-series analysis of the significance of different network sources for female candidates. The shaded area represents the 95% bootstrap CI for the trends.

Extending our analysis, we provide in this subsection a detailed breakdown of the collective contribution of all network sources. This detailed assessment offers insight into the strategic development of professional networks by dissecting the importance of each network source over time, alongside the observation of gender-specific differences. Figure 11 presents an overview of the weighted contribution of each network source, and Figures 12 and 13 detail each contribution to the network over time for men and women, respectively.

Educational bonds consistently played a crucial role in professional advancement. Figure 11 shows that the EDU networks contributed a weighted average of 26% for men and 23% for women, indicating a slightly higher significance for men. Over time, as shown in Figures 12 and 13, the reliance on these networks remained relatively stable for both genders, with only minor fluctuations, indicating the long-lasting value of educational backgrounds in securing board positions.

In employment networks, the weighted contributions showed clear gender differences. CE networks contributed more for men (at 24%) but significantly less for women (at 20%), with *p* <0.001, while PE networks showed relatively similar contributions, with 23% for men and 22% for women, underscoring their importance for both genders. Over time, the trend in the CE networks for men revealed a general decline, beginning at 26% and ending at 15% (*p* <0.001), suggesting a decreasing dependence on this network source. In contrast, women demonstrated an increasing dependence on CE networks, with contributions increasing significantly. This result highlights the fact that women directors had been chosen based on their CE achievements at a higher rate than men, who had been promoted with a higher probability of their previous employment.

The analysis of social engagement networks also revealed distinct gender differences in networking strategies. Women exhibited a greater dependence on current and past social networks, with weighted contributions of 18% and 16%, respectively, toward their paths to board positions, in contrast to the respective weighted contributions of 14% and 13% observed for men (*p* <0.001 for both CSE and PSE). This disparity suggests that women benefited from and relied more heavily on active social bonds when advancing to corporate boards. Meanwhile, although social networks were valuable for men, their impact was less pronounced than other factors such as EDU and employment networks. Over time, the contribution of social engagement for women remained relatively stable, fluctuating between 15% and 20%, while it remained mostly below 15% for men. This stability and disparity underscore the different ways in which men and women benefit from social networks during their career advancement.

Across both genders, EDU and employment networks were identified as more crucial than social engagement to advance to board positions. However, the distribution of reliance on these network sources differed significantly between genders. Although men demonstrated a more pronounced dependence on EDU and employment networks, women exhibited a more uniform distribution of dependence between EDU, employment, and social engagement networks. This balanced approach suggests that actively cultivating broad, multifaceted networks—including social ties—is crucial for women’s professional advancement. In contrast, men may rely more heavily on traditional professional networks. This variation underscores the need for women to invest in diverse networking strategies to navigate the complexities and challenges of reaching top executive positions.

### Influence of network centralities

#### Model results across different network centralities

**Figure 14:**
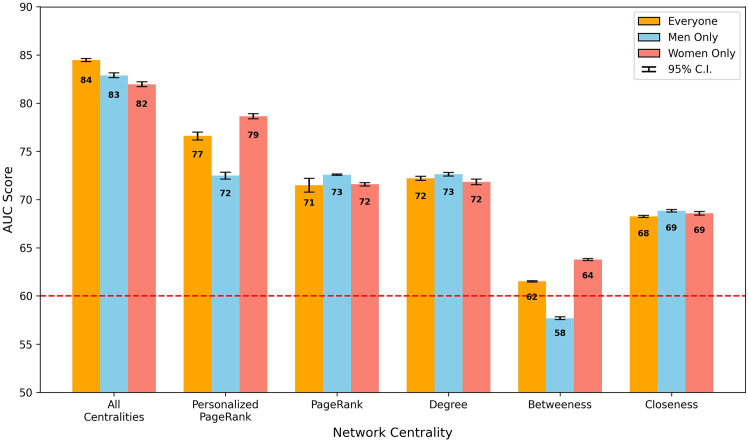
Network centrality measure AUC by group Bar graph showing the model’s performance, measured by AUC scores, across different network centrality measures. The figure comparatively illustrates the predictive power (with a 95% confidence interval) of five different centrality measures in determining board director appointments, with a focus on gender disparities. The dashed line at AUC = 0.60 represents the minimum threshold for a single network centrality’s influence to be considered significant.

Building on the previous exploration of network sources, we now address the critical role that network centrality measures play on the path to board membership. The impact of network centrality measures on the likelihood of attaining board director positions is shown in Figure 14, in which each bar presents the results of fine-tuned models of different centrality measures with a confidence interval of 95%. The analyses have also been divided into three groups for in-depth exploration: a collective assessment of all candidates and separate detailed evaluations for male and female candidates specifically.

Degree, PageRank, and closeness centrality measures showed a balanced impact on board appointments between genders, with nearly identical AUC scores for men and women (0.72 for both degree and PageRank, with *p* <0.001, and 0.69 for closeness, with *p* <0.05). This result suggested an equitable benefit from the breadth and quality of network connections and the efficiency of network reach between genders.

However, distinctions became evident in the PPR and betweenness centrality measures. PPR, which focuses on direct connections with incumbent board members, highlighted a notable variance, with women achieving a higher AUC of 0.79 compared to men’s AUC of 0.72 (*p* <0.001). This effect indicated that exposure of female executives to other directors, as they were the ones who propagated influence, had a higher likelihood of leading to an appointment as a director than for men. The difference was more pronounced in betweenness centrality, in which women significantly outperformed men (AUC score of 0.64 for women versus 0.58 for men, with *p* <0.001). This result suggests that either women position themselves as network bridges or, due to there being fewer women in executive positions, connections to them are leveraged to a greater extent when appointing new board members.

#### Temporal retention of centralities

Retention patterns for network centralities show how structural positions are carried forward in the model memory. Higher values of the retention index indicate that information from a centrality is preserved more strongly across lags.

Across the paired sample (Table S10), the average ranking is stable: PPR (2.171) and PageRank (1.834) are highest, degree (1.509) and closeness (1.400) are intermediate, and betweenness is lowest (0.806). Retention is front-loaded across measures (about 55%–60% of the remembered mass in the first five lags), but the decay profiles differ sharply: degree declines by 53% from the first to the last lag and closeness declines by 48%, whereas PageRank and PPR decline more gradually (around 35%). Betweenness remains low and declines steadily.

**Figure 15:**
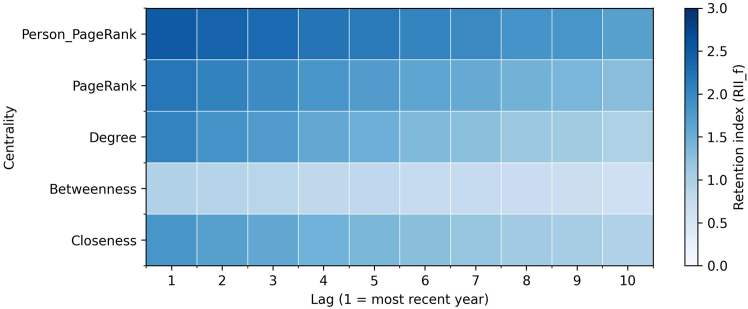
Retention analysis network centralities for men Heatmap of the retention index across 10 lags for 5 network centralities for men. Retention is concentrated in the most recent part of directors’ histories, with personalized PageRank and PageRank preserved most strongly, followed by degree and closeness. Betweenness is retained least, and all centralities decay rapidly as the model looks further back.

**Figure 16:**
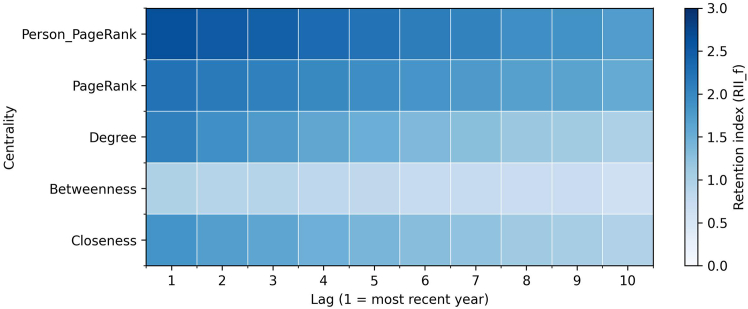
Retention analysis network centralities for women Heatmap of the retention index across 10 lags for 5 network centralities for women. Retention is concentrated in the most recent part of directors’ histories, with personalized PageRank and PageRank preserved most strongly, followed by degree and closeness. Betweenness is retained least, and compared with men, women show somewhat stronger persistence in degree and closeness alongside visibility from PageRank measures.

Figures 15 and 16 report gender-specific patterns. For men, PPR (2.091) and PageRank (1.694) dominate, followed by degree (1.446) and closeness (1.347), with the lowest betweenness (0.782). Degree and closeness are concentrated in early lags and fade quickly; PageRank measures persist longer. For women, PPR (2.179) and PageRank (1.880) are again strongest, with degree (1.464) and closeness (1.364) next, and betweenness lowest (0.790). Two features stand out relative to men: (1) higher average retention for PageRank (+0.186) and PPR (+0.088) and (2) slower decay for PageRank (a 30% drop from the first to the last lag for women versus 41% for men). Differences in degree and closeness are smaller, and their rapid decay is similar between genders.

Overall, centrality retention reveals a short-lived pattern for direct connections and proximity (degree and closeness) and a more persistent pattern for visibility within influential neighborhoods (PageRank and PPR). The gender contrast is concentrated in PageRank: women show both higher levels and a slower decline, indicating that sustained visibility remains present for longer in women’s board trajectories.

#### Breakdown of network centrality contribution

**Figure 17:**
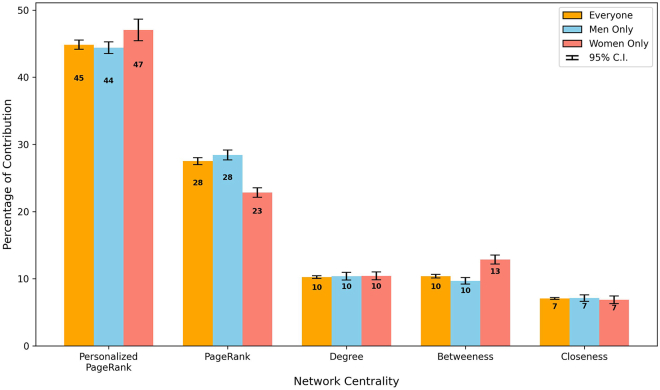
Network centrality measure contribution breakdown Breakdown of the weighted contribution of different network centrality measures to the likelihood of board appointments, including a 95% confidence interval of the bar value. This reveals the comparative significance of various roles in shaping boardroom paths.

**Figure 18:**
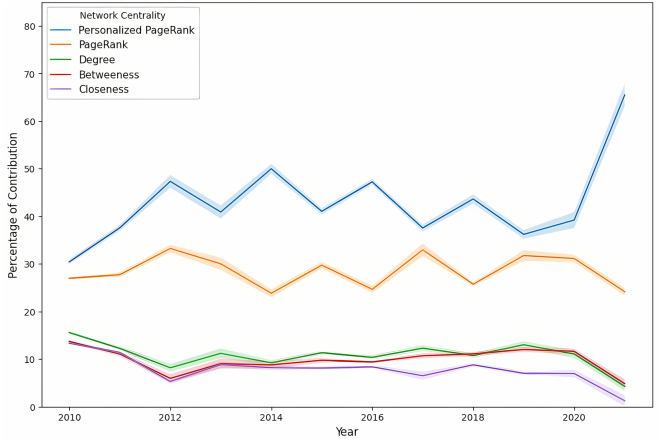
Time-series analysis of the significance of different network centralities for men Time-series analysis of the significance of different network centralities for male candidates. The shaded area represents the 95% bootstrap CI for the trends.

**Figure 19:**
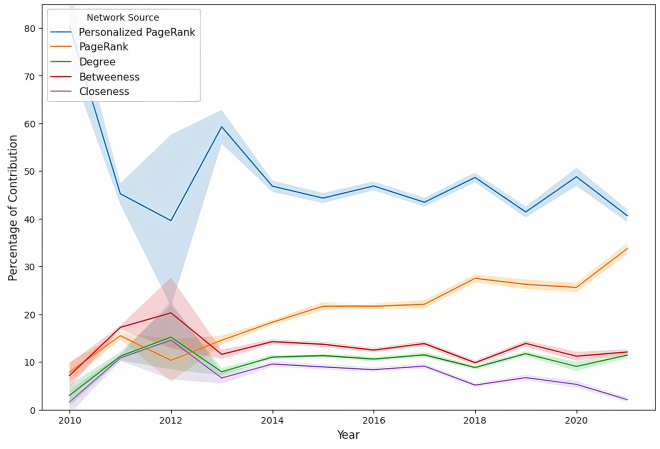
Time-series analysis of the significance of different network centralities for women Time-series analysis of the significance of different network centralities for female candidates. The shaded area represents the 95% bootstrap CI for the trends.

Following our previous exploration of individual network centrality measures, this segment broadens our analysis to comprehensively assess their collective impact. We dissected the weighted contribution of each centrality and examined their influential fluctuations over time, providing a differentiated picture of influence distinguished by gender. Figure 17 illustrates the weighted contribution of each centrality measure, providing a visual representation of their individual and collective contributions. Figures 18 and 19 further detail these contributions, parsing trends over time for men and women, respectively, to enhance understanding of the gendered nuances in the powers of the professional network.

PPR was the most significant centrality measure, with weighted contributions of 47% for women and 44% for men (*p* <0.01), as shown in Figure 17. Although this difference was not statistically significant under a 95% confidence interval, PPR remained the main network centrality for both genders. This finding suggests that building direct connections with current board members is a key strategy to achieve board appointments, regardless of gender.

For PageRank centrality, men exhibited a weighted contribution of 28%, whereas it was initially much lower for women at 23% (*p* <0.001). Figures 18 and 19 show that, while the PageRank contribution for men remained stable near 28%, there was a noticeable increasing trend for women, starting near 10% and increasing to 30%. This trend may have suggested that as more women were appointed to board positions, their networking strategies became more similar to those of their male counterparts, emphasizing the importance of knowing key influential leaders. This alignment could indicate a shift toward more gender-inclusive networking practices in corporate boards, in which the traditional barriers that have differentiated the networking strategies of men and women are becoming less pronounced. The increasing importance of PageRank for women underscored the growing recognition that both knowing important people and making direct strategic connections (as highlighted by the slightly higher significance of PPR for women) were crucial for navigating the path to board positions.

Degree centrality, which indicates the number of direct connections of an individual, and closeness centrality, which represents the efficiency of reaching out within a network, both showed a consistent level of contribution at approximately 10% for degree centrality and 7% for closeness centrality for all groups. This consistency implied that the foundational reach of one’s network was of equal importance for both men and women in their pursuit of board positions.

Betweenness centrality highlighted a significant gender disparity, with women demonstrating a 13% contribution, notably higher than the 10% contribution for men (*p* <0.001). As discussed previously, this effect can be attributed to the relative scarcity of women in senior management roles, which naturally placed them as crucial intermediaries, thus producing a higher betweenness score.

These analyses highlighted the shifting landscape of network centrality over time and its varied impact on board director appointments. Although some centrality measures, such as degree and closeness, maintained a steady level of importance, others, such as PPR, PageRank, and betweenness, exhibited notable gender-specific trends. These observations not only illuminated the evolving dynamics of professional networks but also emphasized the strategic importance of navigating these roles in advancing to board positions.

### Insights from gender-specific PPR

One critical question at the heart of our exploration was as follows: does gender influence the way individuals assist each other in board member appointments? To this end, we applied PPR with gender-specific propagation, targeting existing female and male directors to assess the strength of the same-gender support within the broader network context. The PPR algorithm quantified the level of directed support that women and men had received from other female or male board members.

**Figure 20:**
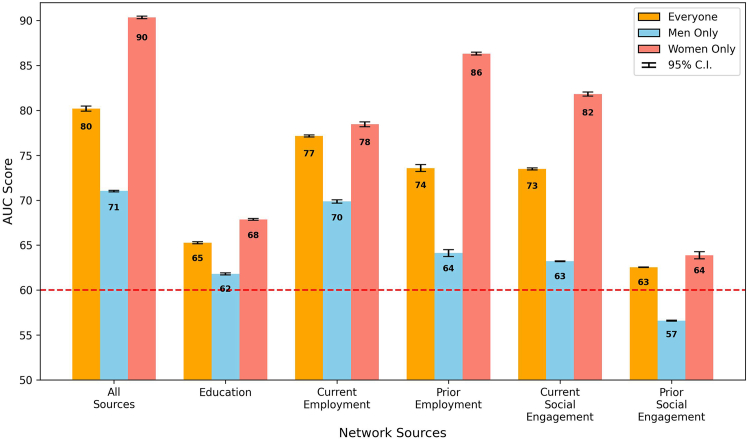
Analysis of female-to-female support Analysis of the impact of female-to-female support within professional networks on board appointments, using gender-specific (female) personalized PageRank. The graph highlights the substantial role of existing female board members in promoting women’s ascent to leadership roles. We include the 95% confidence intervals of the values.

The results of the gender-specific PPR analysis, shown in Figure 20, demonstrated a notable pattern: in all network sources examined, female directors significantly improved the progression of other women to board positions. An aggregated AUC score of 0.90 for women, considering all network sources, underscores the strong support network among women within the corporate sector. In further investigation, we found that women outperformed men in AUC scores in each network source (with *p* <0.001 for every network source), particularly in employment and CSE networks. This observation indicates that women in leadership positions are crucial in creating environments conducive to the advancement of other women, extending from current to past employment ties and into the realm of social engagements. These findings consistently emphasized the unique and powerful support that women provided each other in professional settings.

**Figure 21:**
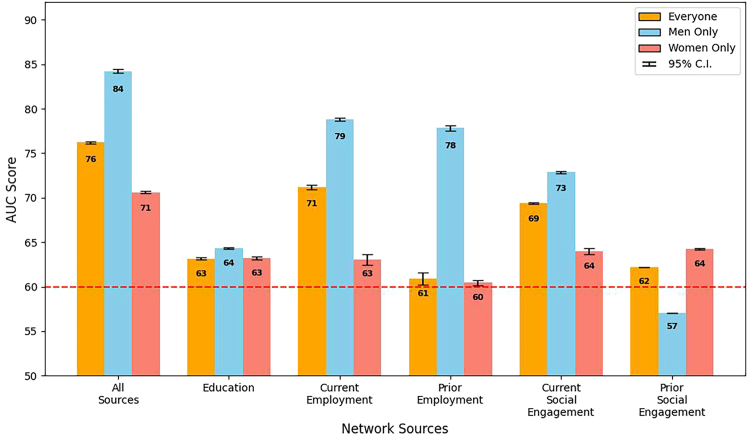
Analysis of male-to-male support Analysis of male-to-male support in professional networking and its effect on board director appointments, using gender-specific (male) personalized PageRank. The graph illustrates the influence of male directors on the professional advancement of their male counterparts. We include the 95% confidence intervals of the values.

The gender-targeted PPR analysis centered on male directors also revealed a significant and distinct pattern of influence within the professional network. As illustrated in Figure 21, male candidates experienced a strong level of support from male directors, reflected in an AUC score of 0.84 when all sources were aggregated. This score, while indicative of robust support, did not reach the level of impact seen in the women-to-women network, suggesting nuances in the way support was extended and leveraged within gendered networks.

The variance observed leads to an intriguing insight: women, in fact, derive greater benefit from the support of other women within their professional networks toward securing board roles. This gender-specific solidarity among female directors can play a crucial role in countering gender imbalances in the boardroom and can inform strategies and policies aimed at enhancing gender diversity in corporate leadership.

These findings demonstrate the impact of gender on networking dynamics in corporate progression, particularly focusing on the support women receive from female directors on their path to board appointments. Societies of female directors and executives, therefore, have an oversized role in reducing the structural inequalities that are currently observed in the market.

## Discussion

The comprehensive analysis presented up to now has explored various aspects of how professional networks influence board director appointments, with a particular focus on gender disparities. Our findings have revealed that while networks are instrumental in career progression, their impact is distinctly moderated by gender, with significant implications for women’s advancement to leadership positions within publicly traded companies.

Our examination of various network sources and centrality measures shows, first, the structural advantages inherent within professional networks, and second, the gender-specific dynamics that influence these benefits. For example, our PPR analysis revealed that female directors have the ability to significantly improve the progression chances of other women, indicating a potent network effect through which incumbent female board members play a role in supporting the ascension of their female peers. This gender-specific solidarity is not as pronounced in male networks, suggesting a unique leverage point that could be used to enhance gender diversity at the highest levels of corporate governance. This pattern is consistent with findings in other domains. For example, Yang et al.^62^ demonstrated that the success of women in leadership is strongly predicted by the gender composition and communication patterns of their networks. Our results extend this insight to the context of the corporate board, suggesting that women’s advancement is particularly sensitive to the structure and quality of their professional ties.

Furthermore, the differential impact of the network sources points to the need for a more detailed understanding of how these networks function separately and in concert. For women, social engagement networks emerged as particularly beneficial, emphasizing the importance of informal networking opportunities alongside formal professional networks. This finding aligns with broader discussions regarding the “invisible barriers” women face in the workplace, suggesting that improving both access to social networks and the efficacy of social networks could be a strategic focus for companies aiming to improve gender diversity in their leadership ranks.

In addition, our results highlight that the effects of networks on board appointments evolve, reflecting broader shifts in corporate culture and governance practices. Understanding these dynamics is crucial for informing strategies aimed at improving gender diversity on boards. Examples could include targeted recruitment practices that actively value the capital of women’s networks, mentoring initiatives that strengthen women’s professional ties, and policies that encourage incumbent directors to sponsor and support other women. Previous research underscored the importance of such network-based mechanisms for the advancement of women,^17^^,^^62^ and our findings reinforce that strengthening women’s professional connections offers a practical way to promote greater inclusivity in corporate governance.

In addition to predictive performance, our analysis of the LSTM’s retain-forget behavior shows how the model internally weighs information. Recent information is emphasized most strongly, while EDU and employment are preserved more than social engagement, and PageRank-based measures are sustained longer than degree, closeness, or betweenness. We also find that informal activities are retained more visibly in women’s trajectories. This provides further evidence that both the timing and the type of network information matter to understanding board pathways.

### General conclusions

Our study analyzed how professional networks and gender disparities have intersected in the appointment of board directors within Canadian public companies. With a detailed analysis of more than 19,000 people in 772 companies from 2000 to 2022, our findings highlighted the effect of the “glass ceiling.”

Our analysis started from a sample in which we matched women with men on their career, sector, and organization-type paths, as well as demographic characteristics, successfully matching 97% of women. This indicates that the women were as qualified as the men. However, the results of our models told a different story about their likelihood of board appointment. Overall, male data were more predictive, both in terms of network source and network position, and when looking closer at the different sources or positions, we found clear imbalances.

In network sources, men’s EDU and employment networks stood out with the greatest predictive value, each contributing 23%–26% to the likelihood of board appointments, while for women, all five network sources contributed more or less equal amounts. This means that women needed to be prominent in social activities as well as in their professional ones to achieve the same results as their male counterparts.

In network position, men and women were equal in terms of degree, PageRank, and closeness, but women’s PPR and betweenness had a higher weight than men’s, showcasing that women needed to position themselves differently regarding current board members to increase their likelihood of board appointment. Finally, the PPR results told an important story. Male directors influenced men’s succession and female directors influenced women’s in all network sources except one: PSEs. For women, the influence of a director (of any gender) in this context was vital, indicating their need to maintain long-term relationships with people already in the positions these up-and-coming professionals desire to achieve.

However, there is a complementary, much more stark interpretation of these results: despite sharing similar demographics and career trajectories, women needed to foster more dense and influential networks than men to reach equivalent influential positions.

This systemic issue, rooted in the entrenched practices of corporate networking, highlights a profound gender influence within professional circles. Our gender-specific PPR analysis revealed that networks comprising female directors offer more substantial support for women aiming for board positions than networks of their male counterparts.

The implications of our findings offer actionable insights for improving corporate governance structures and policies. Initiatives at both organizational and national levels should aim to institutionalize mentoring and networking platforms that promote diversity and inclusion, particularly at the senior management level.

We hope that our research, grounded in empirical bases, contributes significantly to understanding the dynamics of professional networks and gender disparities at the highest levels of corporate governance. It has also provided a unique methodology with which to study causal effects over complex semi-structured data using the latest advances in deep learning. Traditional data treatment tools in causal learning complement this roster of tools, allowing a detailed empirical analysis that is extendable to controlled data, if such data were to be available, or observational data using propensity scores, as we used in this work. However, while our matching design strengthens causal interpretation, it cannot eliminate all unobserved confounders. As with any observational study, therefore, our conclusions about causality should be regarded as suggestive rather than definitive.

### Future directions

Despite the richness of our dataset, our study opens new questions. Our specific focus on the Canadian corporate environment may have restricted the generalizability of our findings to other contexts, although we can be confident that this extends to most countries in the Western world, given their similar legal environments. Additionally, as is the case in all observational studies, relying solely on publicly disclosed information may not have captured the full spectrum of network dynamics. Future research directions should include incorporating longitudinal data to observe changes over time and exploring the intersectionality of gender with other demographic factors, which could provide more detailed insight into barriers to diversity in leadership roles.

In closing, our study underscored the critical role of professional networks in shaping gender diversity within corporate boardrooms. As the corporate world evolves, there is a continuous need for scholarship and practice to investigate and innovate the structures that govern corporate leadership appointments. Achieving gender parity in boardrooms transcends compliance to become a strategic imperative that enriches corporate governance and organizational performance. Our research has set the stage for further exploration into this complex field, advocating for concerted efforts to forge inclusive and equitable leadership pathways for all.

## Resource availability

### Lead contact

Requests for further information and resources should be directed to and will be fulfilled by the lead contact, Prof. Cristian Bravo (cbravoro@uwo.ca).

### Materials availability

This study did not generate new unique reagents.

### Data and code availability

• The BoardEx data used in this study are subscription based and are accessed through the WDRS repository and other alternatives; its terms of service prohibit redistribution of the raw data, but subscribers with valid access credentials may obtain the full dataset directly from the provider. Instructions for constructing the dataset and the computer code to do so are available in our repositories.
• Our source code is available at GitHub and has been archived at Zenodo.^63^

## Acknowledgments

M.D. and C.B. acknowledge the support of the 10.13039/501100000038NSERC Discovery Grant program (RGPIN-2020-07114 and RGPIN-2020-06667). C.B. also acknowledges the support of the SSHRC Insight program (435-2025-1038). M.O. acknowledges the support of the Icelandic Research Fund (IRF) (grant number 228511-051). This research was carried out, in part, thanks to funding from the 10.13039/501100001804Canada Research Chairs program (CRC-2018-00082) and the Ontario Graduate Scholarship program from the Government of Ontario. This work was enabled in part by the support provided by Compute Ontario (https://www.computeontario.ca), Calcul Québec (https://www.calculquebec.ca), and the Digital Research Alliance of Canada (https://www.alliancecan.ca/en).

## Author contributions

Conceptualization, Y.Z., M.O., M.D., and C.B.; methodology, Y.Z., M.O., M.D., and C.B.; software, Y.Z. and W.C.; formal analysis, Y.Z. and W.C.; investigation, Y.Z. and W.C.; data curation, Y.Z. and W.C.; writing – original draft, Y.Z.; writing – review & editing, M.O., M.D., and C.B.; visualization, Y.Z.; validation, M.O., M.D., and C.B.; supervision, M.O., M.D., and C.B.; resources, M.D. and C.B.; funding acquisition, M.D. and C.B.; project administration, C.B.

## Declaration of interests

Y.Z. has taken a position recently at CIBC in Canada. The work occurred with his Western affiliation and not with CIBC, so it is not included in the work.

## Declaration of generative AI and AI-assisted technologies in the writing process

During the preparation of this work, Y.Z. used ChatGPT to assist with formatting checks, tense consistency, grammar checks, and alignment with journal publication requirements. After using this tool/service, the authors reviewed and edited the content as needed and assume full responsibility for the content of the published article.

## Supplemental information

Document S1. Tables S1–S10, Notes S1–S4, and Methods S1 and S2

Document S2. Article plus supplemental information
