# Supplementary material for: Unveiling gender disparities in corporate board career paths using deep learning
Source: Patterns (N Y). 2026 Mar 12;7(4):101495. doi: 10.1016/j.patter.2026.101495 (PMC13083631; doi:10.1016/j.patter.2026.101495)
Supplement: Document S1. Tables S1–S10, Notes S1–S4, and Methods S1 and S2 [file mmc1.pdf]

Patterns, Volume *7*

Supplemental information

Unveiling gender disparities in corporate board career paths using deep learning

Yuhao Zhou, Wenhao Chen, María Óskarsdóttir, Matt Davison, and Cristián Bravo

Supplementary Notes

Note S1: Job categories

In the data cleaning and preparation process, we consolidated over 6,000 uncleaned role names into 18 distinct categories to reduce variation and ensure consistency across the dataset. Given the heterogeneity of corporate structures, job titles such as "associate" or "manager" may not carry identical meanings across firms. Therefore, complete one-to-one comparability was not feasible. To improve robustness and maintain interpretability, we aggregated titles into standardized categories that mitigate firm-level differences in naming practices while preserving meaningful distinctions in career trajectories. The following is the final list of the categorized positions used in our study.

- VP (Vice President)
- Supervisory Director
- Advisor
- C_suite
- Senior VP (Vice President)
- Executive Director
- President
- Manager
- Partner
- Associate
- Director
- Founder
- Officer
- Head
- Executive
- Managing Director
- Other
- Various Positions

Each category represents a specific level or type of role within the organizations studied, ranging from executive positions like 'C_suite' and 'Managing Director' to more generalized categories such as 'Other' and 'Various Positions', which are provided by BoardEx.

Note S2: Index categories

This appendix provides a detailed list of the company indexes used in the study, as provided by BoardEx. These indexes are integral to understanding the various market segments and economic environments of which the companies analyzed are part. Below is the list of indexes as they appear in BoardEx's data dictionary:

- S&P/TSX 60; S&P/TSX Composite
- S&P/TSX Composite
- S&P 500
- S&P Mid Cap 400
- S&P 100; S&P 500
- S&P 100; S&P 500; Dow Jones Industrial Average
- S&P 100; S&P 500; NASDAQ 100; Dow Jones Industrial Average
- S&P Small Cap 600
- S&P 500; NASDAQ 100
- S&P 100; S&P 500; NASDAQ 100
- S&P 500; Dow Jones Industrial Average
- NASDAQ 100

- ASX All Ordinaries
- FTSE AIM
- S&P Small Cap 600; MDAX; TecDAX
- S&P/ASX 300; S&P/TSX Composite; ASX All Ordinaries
- NASDAQ 100; Hang Seng; Hong Kong Hang Seng China Enterprises
- No Index

Each index listed represents a different grouping of companies based on various criteria such as market capitalization and listed location (if in multiple regions).

Note S3: Nationality

The following table provides a categorization of nationalities grouped by geographical regions, as used in the study.

Grouped nationality	Nationality
Europe	Spanish, British, German, Belgian, Irish, Swedish, Italian, French, Norwegian, Dutch, Greek, Finnish, Portuguese, Danish, Swiss, Romanian, Austrian, Icelander, Serbian, Polish, Croatian
East Asia	Chinese, Japanese
South Asia	Indian, Pakistani
Southeast Asia	Vietnamese, Malaysian, Singaporean, Indonesian, Cambodian
Middle East	Israeli, Lebanese, Emirian, Saudi, Egyptian
Africa	South African, Central African, Nigerien, Ethiopian, Ugandan, Ghanaian, Mauritanian, Congolese (DR Congo), Nigerian, Namibian, Zimbabwean
America	American
Canada	Canadian
Central America	Mexican
South America	Argentine, Peruvian, Brazilian, Colombian, Chilean, Uruguayan, Venezuelan
Caribbean	Trinidadian/Tobagonian, Guyanese
Oceania	Australian, New Zealander

Note S4: Hyperparameters

The model's hyperparameters including the learning rate, hidden layer dimensions, and training epochs. The fine-tuned value setup is outlined as below:

- Learning Rate: 0.01, 0.05, 0.1
- Hidden Layer Dimensions: 64, 128, 256
- Training Epochs: 50, 100, 150

Supplementary Methods

Method S1: Optimal Matching

The OM distance $d(i,j)$ between the sequences S_i and S_j can be expressed as:

$$d(i,j) = \min\left(\sum_{k=1}^{n} c_k \cdot \delta_k\right)$$

where:

- c_k represents the cost of the k-th operation (insertion, deletion, or substitution),
- δ_k is an indicator function that is 1 if the k-th operation is performed and 0 otherwise,
- n is the total number of operations needed to transform sequence S_i into sequence S_j.

Consider two general sequences S_i and S_j:

$$S_i = [s_{i1}, s_{i2}, \ldots, s_{im}]$$
$$S_j = [s_{j1}, s_{j2}, \ldots, s_{jn}]$$

To transform S_i into S_j, we may need to perform the following types of operation:

- **Insertion**: Insert an element into S_i. For example, if we insert the element s into S_i at position k, the cost is $c_{\text{insert}}(s,k)$.
- **Deletion**: Delete an element from S_i. For example, if we remove the element s_{ik} from S_i, the cost is $c_{\text{delete}}(s_{ik},k)$.
- **Substitution**: Substitute an element in S_i with an element from S_j. For example, if we substitute element s_{ik} in S_i with element s_{jk} from S_j, the cost is $c_{\text{substitute}}(s_{ik}, s_{jk})$.

Thus, the OM distance $d(i,j)$ can be expressed more generally as:

$$d(i,j) = \min\left(\sum_{k=1}^{n} \left(c_{\text{insert}}(s,k) \cdot \delta_{\text{insert}} + c_{\text{delete}}(s_{ik},k) \cdot \delta_{\text{delete}} + c_{\text{substitute}}(s_{ik}, s_{jk}) \cdot \delta_{\text{substitute}}\right)\right)$$

where $\delta_{\text{insert}}, \delta_{\text{delete}}, \delta_{\text{substitute}}$ are indicator functions that are 1 if the respective operation is performed and 0 otherwise.

To ensure comparability across these matrices, we normalized each dissimilarity score, setting the lowest value at 0 and the highest at 1. A dissimilarity score of 0 indicates identical paths, implying that no transformation costs or operations are required, a scenario typically observed in self-comparisons. In contrast, the maximum value of 1 represents the most dissimilar pair, indicating the highest costs required for the sequence transformation.

Method S2: Significance Test

This appendix reports significance testing for the differences presented in Section ”Results”. Although the main text emphasizes substantive patterns and effect sizes, here we formally assess whether observed differences are statistically significant. All tests follow a unified methodology, described in the following, and the results are reported in tabular form for each subsection of ”Results”.

We approximate statistical significance directly from the performance means reported and 95% confidence intervals (CI). Let $\hat{\mu}$ denote the reported mean and (L, U) the corresponding CI bounds. The standard error (SE) is obtained as follows:

$$SE \;=\; \frac{U - L}{2 \times 1.96}.$$

For each comparison of two groups i and j, we compute the difference:

$$\Delta = \hat{\mu}_i - \hat{\mu}_j,$$

with standard error of the difference:

$$SE(\Delta) = \sqrt{SE_i^2 + SE_j^2}.$$

The test statistic is then

$$z = \frac{\Delta}{SE(\Delta)}.$$

The Two-sided p-values are obtained from the standard normal distribution. This procedure is equivalent to testing whether the differences in reported means are significantly different from zero given their uncertainty estimates.

As the matched dataset comprises 4,228 female–male pairs (8,456 individuals), the reported CIs are narrow, allowing precise inference. We adopt conventional thresholds: $p < 0.10$ (†), $p < 0.05$ (*), $p < 0.01$ (**).

Each subsection of this appendix corresponds to the parallel subsection in the main text (Results) and presents tables with Δ, z, and p-values for all group comparisons.

Supplementary Tables

Table S1: Correlation matrix for Network

Table S1: Correlation matrix for Network

Score	EDU					CE					PE				
	PR	PPR	Betw	Clos	Deg	PR	PPR	Betw	Clos	Deg	PR	PPR	Betw	Clos	Deg
**EDU_PageRank**	1.000	0.878	0.650	0.772	0.859	-0.146	-0.122	0.028	-0.125	-0.096	-0.001	0.019	0.052	-0.064	0.014
**EDU_Person_PageRank**	0.878	1.000	0.617	0.649	0.789	-0.140	-0.072	0.058	-0.118	-0.112	0.036	0.091	0.075	-0.016	0.036
**EDU_betweeness**	0.650	0.617	1.000	0.371	0.601	-0.039	-0.024	0.026	-0.028	-0.028	0.042	0.054	0.059	0.012	0.040
**EDU_closeness**	0.772	0.649	0.371	1.000	0.676	-0.182	-0.160	0.024	-0.155	-0.109	-0.047	-0.029	0.030	-0.117	-0.015
**EDU_degree**	0.859	0.789	0.601	0.676	1.000	-0.054	-0.037	0.038	-0.026	-0.041	0.061	0.072	0.066	0.027	0.054
**CE_PageRank**	-0.146	-0.140	-0.039	-0.182	-0.054	1.000	0.912	0.218	0.843	0.684	-0.008	-0.040	-0.026	0.258	-0.022
**CE_Person_PageRank**	-0.122	-0.072	-0.024	-0.160	-0.037	0.912	1.000	0.280	0.749	0.521	0.010	0.034	0.002	0.251	-0.034
**CE_betweeness**	0.028	0.058	0.026	0.024	0.038	0.218	0.280	1.000	0.141	0.125	0.082	0.116	0.118	0.097	0.060
**CE_closeness**	-0.125	-0.118	-0.028	-0.155	-0.026	0.843	0.749	0.141	1.000	0.645	-0.003	-0.033	-0.015	0.278	-0.002
**CE_degree**	-0.096	-0.112	-0.028	-0.109	-0.041	0.684	0.521	0.125	0.645	1.000	0.014	-0.044	-0.037	0.236	0.094
**PE_PageRank**	-0.001	0.036	0.042	-0.047	0.061	-0.008	0.010	0.082	-0.003	0.014	1.000	0.936	0.552	0.698	0.845
**PE_Person_PageRank**	0.019	0.091	0.054	-0.029	0.072	-0.040	0.034	0.116	-0.033	-0.044	0.936	1.000	0.545	0.637	0.774
**PE_betweeness**	0.052	0.075	0.059	0.030	0.066	-0.026	0.002	0.118	-0.015	-0.037	0.552	0.545	1.000	0.227	0.438
**PE_closeness**	-0.064	-0.016	0.012	-0.117	0.027	0.258	0.251	0.097	0.278	0.236	0.698	0.637	0.227	1.000	0.563
**PE_degree**	0.014	0.036	0.040	-0.015	0.054	-0.022	-0.034	0.060	-0.002	0.094	0.845	0.774	0.438	0.563	1.000
**CSE_PageRank**	0.114	0.157	0.123	0.094	0.118	0.004	0.028	0.044	0.023	0.019	0.053	0.071	0.043	0.052	0.073
**CSE_Person_PageRank**	0.104	0.171	0.119	0.081	0.105	-0.011	0.030	0.046	0.007	0.001	0.037	0.070	0.041	0.038	0.050
**CSE_betweeness**	0.064	0.090	0.079	0.044	0.067	0.008	0.023	0.020	0.016	0.011	0.030	0.042	0.023	0.033	0.040
**CSE_closeness**	0.126	0.166	0.121	0.113	0.134	0.011	0.030	0.039	0.031	0.034	0.058	0.072	0.045	0.059	0.085
**CSE_degree**	0.104	0.132	0.108	0.085	0.114	0.013	0.028	0.029	0.027	0.020	0.039	0.051	0.035	0.044	0.049
**PSE_PageRank**	0.123	0.165	0.150	0.093	0.128	-0.023	-0.001	0.032	-0.005	-0.006	0.060	0.076	0.057	0.045	0.078
**PSE_Person_PageRank**	0.115	0.176	0.146	0.081	0.116	-0.030	0.002	0.030	-0.013	-0.016	0.041	0.069	0.051	0.032	0.055
**PSE_betweeness**	0.087	0.113	0.110	0.056	0.092	-0.012	0.001	0.014	0.002	-0.003	0.034	0.043	0.033	0.024	0.045
**PSE_closeness**	0.136	0.181	0.138	0.116	0.147	-0.013	0.006	0.030	0.009	0.013	0.076	0.090	0.056	0.068	0.102
**PSE_degree**	0.109	0.144	0.124	0.082	0.120	-0.013	0.002	0.023	0.003	0.001	0.051	0.062	0.047	0.040	0.065

Score	CSE					PSE				
	PR	PPR	Betw	Clos	Deg	PR	PPR	Betw	Clos	Deg
**EDU_PageRank**	0.114	0.104	0.064	0.126	0.104	0.123	0.115	0.087	0.136	0.109
**EDU_Person_PageRank**	0.157	0.171	0.090	0.166	0.132	0.165	0.176	0.113	0.181	0.144
**EDU_betweeness**	0.123	0.119	0.079	0.121	0.108	0.150	0.146	0.110	0.138	0.124
**EDU_closeness**	0.094	0.081	0.044	0.113	0.085	0.093	0.081	0.056	0.116	0.082
**EDU_degree**	0.118	0.105	0.067	0.134	0.114	0.128	0.116	0.092	0.147	0.120
**CE_PageRank**	0.004	0.028	0.044	0.023	0.019	-0.023	-0.030	-0.012	-0.013	-0.013
**CE_Person_PageRank**	0.028	0.030	0.023	0.030	0.028	-0.001	0.002	0.001	0.006	0.002
**CE_betweeness**	0.044	0.046	0.020	0.039	0.029	0.032	0.030	0.014	0.030	0.023
**CE_closeness**	0.023	0.007	0.016	0.031	0.027	-0.005	-0.013	0.002	0.009	0.003
**CE_degree**	0.019	0.001	0.011	0.034	0.020	-0.006	-0.016	-0.003	0.013	0.001
**PE_PageRank**	0.053	0.071	0.043	0.052	0.073	0.060	0.041	0.034	0.076	0.051
**PE_Person_PageRank**	0.071	0.070	0.042	0.072	0.051	0.076	0.069	0.043	0.090	0.062
**PE_betweeness**	0.043	0.041	0.023	0.045	0.035	0.057	0.051	0.033	0.056	0.047
**PE_closeness**	0.052	0.038	0.033	0.059	0.044	0.045	0.032	0.024	0.068	0.040
**PE_degree**	0.073	0.050	0.040	0.085	0.049	0.078	0.055	0.045	0.102	0.065
**CSE_PageRank**	1.000	0.896	0.481	0.738	0.773	0.437	0.391	0.236	0.505	0.367
**CSE_Person_PageRank**	0.896	1.000	0.497	0.695	0.773	0.425	0.429	0.254	0.487	0.370
**CSE_betweeness**	0.481	0.497	1.000	0.353	0.453	0.223	0.222	0.213	0.234	0.198
**CSE_closeness**	0.738	0.695	0.353	1.000	0.665	0.393	0.355	0.206	0.558	0.348
**CSE_degree**	0.773	0.773	0.453	0.665	1.000	0.366	0.344	0.234	0.473	0.394
**PSE_PageRank**	0.437	0.425	0.223	0.393	0.366	1.000	0.910	0.637	0.656	0.841
**PSE_Person_PageRank**	0.391	0.429	0.222	0.355	0.344	0.910	1.000	0.649	0.620	0.823
**PSE_betweeness**	0.236	0.254	0.213	0.206	0.234	0.637	0.649	1.000	0.393	0.617
**PSE_closeness**	0.505	0.487	0.234	0.558	0.473	0.656	0.620	0.393	1.000	0.617
**PSE_degree**	0.367	0.370	0.198	0.348	0.394	0.841	0.823	0.617	0.617	1.000

Table S2: Optimal Model Parameters by Group and Category

Table S2: Optimal Model Parameters by Group and Category

Group	Category	Source/Centrality	Training Epochs	Learning Rate	Hidden Layer Dimensions
Everyone	All	All	100	0.1	64
	Source	EDU	100	0.05	64
		CE	100	0.05	64
		PE	100	0.01	128
		CSE	100	0.05	64
		PSE	150	0.05	64
	Centrality	Personalized PageRank	50	0.05	64
		PageRank	150	0.05	128
		Degree	50	0.05	64
		Betweenness	50	0.01	64
		Closeness	150	0.01	128
Men only	All	All	100	0.1	64
	Sources	EDU	100	0.01	64
		CE	100	0.05	64
		PE	100	0.01	64
		CSE	150	0.05	64
		PSE	100	0.1	64
	Centrality	Personalized PageRank	100	0.05	64
		PageRank	100	0.01	128
		Degree	150	0.01	64
		Betweenness	100	0.01	256
		Closeness	100	0.01	256
Women only	All	All	150	0.1	64
	Source	EDU	50	0.05	64
		CE	100	0.1	64
		PE	150	0.01	64
		CSE	150	0.1	64
		PSE	150	0.05	64
	Centrality	Personalized PageRank	50	0.05	64
		PageRank	50	0.05	64
		Degree	150	0.01	256
		Betweenness	100	0.01	64
		Closeness	150	0.01	256

This table shows the fine-tuned results of hyperparameters across all models. As mentioned in the main body of the paper, we have models from three main groups: “Everyone,” ”Men only,” and ”Women only.” For each group, we trained models to learn the influence of networks by including all network sources and centralities or by using only a single network source or a single network centrality.

Table S3: Significance Tests - Network Source AUC by Group

Table S3: Significance Tests - Network Source AUC by Group

Source	Men mean	Women mean	Diff (M–W)	z-value	p-value
ALL	82.89	81.96	0.93	5.16	$<$0.001
EDU	79.66	76.90	2.76	11.13	$<$0.001
CE	77.64	74.29	3.35	9.42	$<$0.001
PE	76.45	76.04	0.41	1.11	0.269
CSE	57.01	66.93	-9.92	-54.82	$<$0.001
PSE	55.16	63.99	-8.83	-71.49	$<$0.001

Significance levels: $^{\dagger}p < 0.10$, $^{*}p < 0.05$, $^{**}p < 0.01$.

Table S4: Significance Tests - Network Source Contribution Breakdown

Table S4: Significance Tests - Network Source Contribution Breakdown

Source	Men mean	Women mean	Diff (M–W)	z-value	p-value
EDU	26.41	23.14	3.27	7.84	$<$0.001
CE	23.54	20.08	3.46	4.64	$<$0.001
PE	22.90	22.06	0.84	0.92	0.359
CSE	14.37	18.24	-3.87	-7.82	$<$0.001
PSE	12.79	16.48	-3.69	-7.04	$<$0.001

Significance levels: $^{\dagger}p < 0.10$, $^{*}p < 0.05$, $^{**}p < 0.01$.

Table S5: Significance Tests - Network Centrality AUC by Group

Table S5: Significance Tests - Network Centrality AUC by Group

Source	Men mean	Women mean	Diff (M–W)	z-value	p-value
ALL	82.89	81.96	0.93	5.16	$<$0.001
PERSON_PAGERANK	72.49	78.65	-6.16	-27.37	$<$0.001
PageRank	72.58	71.58	1.00	10.33	$<$0.001
DEGREE	72.62	71.84	0.78	4.44	$<$0.001
BETWEENESS	57.68	63.78	-6.10	-63.26	$<$0.001
CLOSENESS	68.83	68.58	0.25	2.11	0.035

Significance levels: $^{\dagger}p < 0.10$, $^{*}p < 0.05$, $^{**}p < 0.01$.

Table S6: Significance Tests - Network Centrality Contribution Breakdown

Table S6: Significance Tests - Network Source Contribution Breakdown

Source	Men mean	Women mean	Diff (M–W)	z-value	p-value
PERSON_PAGERANK	44.41	47.05	-2.64	-2.81	0.005
PAGERANK	28.42	22.83	5.59	10.79	$<$0.001
DEGREE	10.37	10.42	-0.05	-0.12	0.904
BETWEENESS	9.68	12.85	-3.17	-7.50	$<$0.001
CLOSENESS	7.12	6.84	0.28	0.73	0.466

Significance levels: $^{\dagger}p < 0.10$, $^{*}p < 0.05$, $^{**}p < 0.01$.

Table S7: Significance Tests - Gender-Specific Personalized PageRank (Men)

Table S7: Significance Tests - Gender-Specific Personalized PageRank (Men)

Source	Men mean	Women mean	Diff (M–W)	z-value	p-value
ALL	71.03	90.35	-19.32	-243.80	$<$0.001
EDU	61.79	67.87	-6.08	-80.16	$<$0.001
CE	69.87	78.45	-8.58	-53.18	$<$0.001
PE	64.12	86.32	-22.20	-107.73	$<$0.001
CSE	63.21	81.81	-18.60	-152.93	$<$0.001
PSE	56.61	63.87	-7.26	-34.82	$<$0.001

Significance levels: $^{\dagger}p < 0.10$, $^{*}p < 0.05$, $^{**}p < 0.01$.

Table S8: Significance Tests - Gender-Specific Personalized PageRank (Women)

Table S8: Significance Tests - Gender-Specific Personalized PageRank (Women)

Source	Men mean	Women mean	Diff (M–W)	z-value	p-value
ALL	84.24	70.60	13.64	106.68	$<$0.001
EDU	64.35	63.18	1.17	11.56	$<$0.001
CE	78.84	63.07	15.77	49.29	$<$0.001
PE	77.83	60.44	17.39	86.02	$<$0.001
CSE	72.86	63.96	8.90	48.38	$<$0.001
PSE	57.06	64.25	-7.19	-297.09	$<$0.001

Significance levels: $^{\dagger}p < 0.10$, $^{*}p < 0.05$, $^{**}p < 0.01$.

Table S9: Retention Indices for Network Sources

Table S9: Retention indices for network sources

Source	lag	RII_f All	RII_i All	RII_f Male	RII_i Male	RII_f Female	RII_i Female
CSE	1	1.202	1.159	1.093	1.063	1.209	1.164
CSE	2	1.093	1.055	0.997	0.970	1.103	1.065
CSE	3	1.069	1.029	0.970	0.941	1.074	1.033
CSE	4	0.962	0.927	0.878	0.852	0.970	0.934
CSE	5	0.942	0.906	0.858	0.829	0.943	0.905
CSE	6	0.833	0.802	0.764	0.738	0.838	0.807
CSE	7	0.811	0.781	0.742	0.716	0.814	0.783
CSE	8	0.710	0.684	0.654	0.631	0.715	0.690
CSE	9	0.700	0.674	0.652	0.629	0.708	0.685
CSE	10	0.608	0.587	0.564	0.546	0.609	0.595
CE	1	2.461	2.426	2.385	2.373	2.308	2.276
CE	2	2.401	2.374	2.332	2.323	2.254	2.232
CE	3	2.352	2.327	2.281	2.274	2.209	2.189
CE	4	2.276	2.261	2.218	2.215	2.142	2.132
CE	5	2.228	2.213	2.170	2.169	2.092	2.084
CE	6	2.149	2.144	2.106	2.109	2.021	2.024
CE	7	2.114	2.108	2.070	2.072	1.986	1.991
CE	8	2.027	2.034	1.998	2.006	1.909	1.925
CE	9	1.999	2.005	1.971	1.977	1.883	1.900
CE	10	1.897	1.916	1.890	1.901	1.796	1.826
EDU	1	2.586	2.526	2.626	2.599	2.546	2.497
EDU	2	2.507	2.457	2.550	2.528	2.470	2.431
EDU	3	2.432	2.382	2.474	2.453	2.399	2.363
EDU	4	2.352	2.313	2.400	2.386	2.322	2.297
EDU	5	2.289	2.249	2.338	2.323	2.258	2.235
EDU	6	2.221	2.193	2.278	2.268	2.191	2.179
EDU	7	2.181	2.149	2.236	2.226	2.150	2.138
EDU	8	2.122	2.103	2.187	2.183	2.093	2.093
EDU	9	2.096	2.072	2.160	2.152	2.066	2.066
EDU	10	2.036	2.025	2.112	2.108	2.011	2.022
PE	1	2.266	2.216	2.176	2.155	2.220	2.177
PE	2	2.181	2.139	2.101	2.085	2.143	2.108
PE	3	2.127	2.085	2.055	2.038	2.098	2.065
PE	4	2.017	1.984	1.957	1.945	1.995	1.971
PE	5	1.966	1.934	1.915	1.904	1.949	1.928
PE	6	1.842	1.817	1.801	1.794	1.826	1.815
PE	7	1.798	1.774	1.765	1.757	1.790	1.778
PE	8	1.670	1.652	1.644	1.640	1.664	1.660
PE	9	1.640	1.619	1.620	1.614	1.636	1.639
PE	10	1.516	1.499	1.497	1.495	1.508	1.515
PSE	1	1.477	1.437	1.237	1.212	1.533	1.484
PSE	2	1.354	1.321	1.130	1.110	1.402	1.360
PSE	3	1.311	1.276	1.091	1.071	1.354	1.313
PSE	4	1.185	1.156	0.983	0.966	1.218	1.184
PSE	5	1.145	1.118	0.948	0.930	1.172	1.137
PSE	6	1.030	1.009	0.851	0.835	1.050	1.022
PSE	7	1.008	0.987	0.826	0.810	1.019	0.991
PSE	8	0.893	0.875	0.736	0.722	0.906	0.883
PSE	9	0.876	0.856	0.722	0.706	0.887	0.866
PSE	10	0.769	0.756	0.640	0.627	0.783	0.768

Values of the Retention Index from the forget gate ($RII^{(f)}$) and the input gate ($RII^{(i)}$) across ten lags, reported for five centralities (Education, Current Employment, Prior Employment, Current Social Engagement, Prior Social Engagement). Higher $RII^{(f)}$ indicates stronger memory of past information, while higher $RII^{(i)}$ indicates stronger intake of new information. Results are shown for the pooled sample and by gender.

Table S10: Retention Indices for Network Centralities

Table S10: Retention indices for network centralities

Measure	lag	RII_f All	RII_i All	RII_f Male	RII_i Male	RII_f Female	RII_i Female
PageRank	1	2.288	2.239	2.169	2.145	2.228	2.181
PageRank	2	2.167	2.126	2.041	2.022	2.138	2.101
PageRank	3	2.076	2.035	1.938	1.919	2.074	2.038
PageRank	4	1.952	1.921	1.812	1.799	1.975	1.948
PageRank	5	1.871	1.841	1.726	1.711	1.916	1.890
PageRank	6	1.754	1.732	1.610	1.601	1.818	1.802
PageRank	7	1.690	1.667	1.539	1.528	1.771	1.755
PageRank	8	1.580	1.566	1.434	1.428	1.678	1.672
PageRank	9	1.535	1.517	1.384	1.376	1.650	1.646
PageRank	10	1.428	1.420	1.284	1.280	1.556	1.562
PersonalizedPageRank	1	2.638	2.590	2.516	2.493	2.635	2.583
PersonalizedPageRank	2	2.516	2.478	2.405	2.388	2.518	2.478
PersonalizedPageRank	3	2.436	2.398	2.328	2.311	2.440	2.402
PersonalizedPageRank	4	2.300	2.273	2.208	2.198	2.309	2.284
PersonalizedPageRank	5	2.228	2.202	2.140	2.129	2.233	2.209
PersonalizedPageRank	6	2.093	2.077	2.022	2.016	2.102	2.091
PersonalizedPageRank	7	2.033	2.017	1.964	1.958	2.041	2.031
PersonalizedPageRank	8	1.897	1.891	1.844	1.844	1.910	1.912
PersonalizedPageRank	9	1.856	1.847	1.807	1.804	1.869	1.874
PersonalizedPageRank	10	1.713	1.716	1.680	1.682	1.730	1.746
Betweenness	1	1.001	0.970	0.956	0.937	0.971	0.944
Betweenness	2	0.937	0.910	0.897	0.881	0.911	0.889
Betweenness	3	0.914	0.887	0.877	0.861	0.892	0.870
Betweenness	4	0.850	0.827	0.820	0.805	0.831	0.814
Betweenness	5	0.830	0.808	0.803	0.788	0.812	0.795
Betweenness	6	0.765	0.747	0.744	0.732	0.750	0.739
Betweenness	7	0.759	0.740	0.736	0.722	0.742	0.731
Betweenness	8	0.693	0.678	0.682	0.671	0.687	0.678
Betweenness	9	0.683	0.666	0.677	0.665	0.680	0.673
Betweenness	10	0.629	0.615	0.625	0.616	0.625	0.623
Closeness	1	1.894	1.850	1.813	1.791	1.843	1.802
Closeness	2	1.754	1.719	1.682	1.664	1.709	1.677
Closeness	3	1.657	1.622	1.587	1.569	1.616	1.585
Closeness	4	1.521	1.494	1.461	1.448	1.485	1.463
Closeness	5	1.434	1.409	1.378	1.365	1.398	1.377
Closeness	6	1.311	1.293	1.266	1.256	1.279	1.266
Closeness	7	1.240	1.222	1.195	1.185	1.208	1.195
Closeness	8	1.131	1.119	1.095	1.088	1.102	1.096
Closeness	9	1.078	1.064	1.043	1.035	1.048	1.045
Closeness	10	0.980	0.973	0.954	0.949	0.954	0.956
Degree	1	2.136	2.082	2.032	2.004	2.066	2.019
Degree	2	1.945	1.901	1.856	1.834	1.887	1.849
Degree	3	1.823	1.781	1.736	1.713	1.765	1.731
Degree	4	1.646	1.614	1.575	1.558	1.599	1.573
Degree	5	1.545	1.514	1.478	1.461	1.498	1.474
Degree	6	1.390	1.367	1.337	1.325	1.350	1.335
Degree	7	1.314	1.291	1.262	1.250	1.274	1.260
Degree	8	1.177	1.162	1.138	1.130	1.146	1.138
Degree	9	1.117	1.101	1.079	1.071	1.085	1.079
Degree	10	0.996	0.986	0.971	0.966	0.973	0.974

Values of the Retention Index from the forget gate ($RII^{(f)}$) and the input gate ($RII^{(i)}$) across ten lags, reported for five centralities (degree, closeness, betweenness, PageRank, Personalized PageRank). Higher $RII^{(f)}$ indicates stronger memory of past information, while higher $RII^{(i)}$ indicates stronger intake of new information. Results are shown for the pooled sample and by gender.
